# Reliability of in vitro data for the mechanistic prediction of brain extracellular fluid pharmacokinetics of P-glycoprotein substrates in vivo; are we scaling correctly?

**DOI:** 10.1007/s10928-025-09963-w

**Published:** 2025-02-08

**Authors:** Daan W. van Valkengoed, Makoto Hirasawa, Vivi Rottschäfer, Elizabeth C. M. de Lange

**Affiliations:** 1 Division of Systems Pharmacology and Pharmacy, Leiden Academic Centre for Drug Research, Leiden University, Leiden, The Netherlands; 2 Mathematical Institute, Leiden University, Leiden, The Netherlands; 3 Korteweg-de Vries Institute for Mathematics, University of Amsterdam, Amsterdam, The Netherlands

**Keywords:** P-glycoprotein, PBPK, CNS, BBB, Pharmacokinetics, IVIVE

## Abstract

Plasma pharmacokinetic (PK) profiles often do not resemble the PK within the central nervous system (CNS) because of blood–brain-border (BBB) processes, like active efflux by P-glycoprotein (P-gp). Methods to predict CNS-PK are therefore desired. Here we investigate whether in vitro apparent permeability (P_app_) and corrected efflux ratio (ER_c_) extracted from literature can be repurposed as input for the LeiCNS-PK3.4 physiologically-based PK model to confidently predict rat brain extracellular fluid (ECF) PK of P-gp substrates. Literature values of in vitro Caco-2, LLC-PK1-mdr1a/MDR1, and MDCKII-MDR1 cell line transport data were used to calculate P-gp efflux clearance (CL_Pgp_). Subsequently, CL_Pgp_ was scaled from in vitro to in vivo through a relative expression factor (REF) based on P-gp expression differences. BrainECF PK was predicted well (within twofold error of the observed data) for 2 out of 4 P-gp substrates after short infusions and 3 out of 4 P-gp substrates after continuous infusions. Variability of in vitro parameters impacted both predicted rate and extent of drug distribution, reducing model applicability. Notably, use of transport data and in vitro P-gp expression obtained from a single study did not guarantee an accurate prediction; it often resulted in worse predictions than when using in vitro expression values reported by other labs. Overall, LeiCNS-PK3.4 shows promise in predicting brainECF PK, but this study highlights that the in vitro to in vivo translation is not yet robust. We conclude that more information is needed about context and drug dependency of in vitro data for robust brainECF PK predictions.

**Supplementary Information:**

The online version contains supplementary material available at 10.1007/s10928-025-09963-w.

## Introduction

Plasma pharmacokinetic (PK) profiles often do not resemble the PK within the central nervous system (CNS), which is mainly caused by the blood–brain-border (BBB) [1]. For compounds that exert their function within the central nervous system (CNS), the BBB poses a challenge, leading to high attrition rates observed for novel CNS drugs [2, 3]. Especially the efflux transporter P-glycoprotein (P-gp) plays a crucial role, as it limits a wide range of drugs in adequately accessing the brain [4, 5]. The complex interplay of passive diffusion, active transporters and intra-brain distribution makes PK profiles in the brain difficult to predict. A mechanistic and integrated understanding of the processes governing BBB transport is crucial for successful predictions of brain PK [6].

Information on the unbound drug PK in the brain extracellular fluid (brainECF), the target site of most CNS drugs, is required to understand the relationship between drug exposure and its effect, and to subsequently optimize therapy. The gold standard to measure this is through microdialysis that can be applied in animal studies, but is restricted in its use in humans due to ethical considerations [7]. Microdialysis studies allow us to improve mechanistic understanding of BBB transport, but animal studies are expensive and extensive (i.e., they are time-consuming and require technical expertise). Also, the use of animals should be restricted where possible. Alternatively, in vitro models and assays hold promise for the prediction of drug penetration into the brainECF in vivo [8]. An example is the transwell permeability assay, used for the study of drug permeability across a monolayer of cells expressing transporters like P-gp [9, 10]. Mechanistic information on the interaction of a drug with a membrane and transporters can be derived from these assays, for prediction of BBB transport in vivo.

Multiple approaches using in vitro data to predict brain distribution of drugs have been published, including for P-gp substrates [11]. Most are focussed on the prediction of the extent of BBB transport, i.e. on Kp_uu,brain_, which is the ratio of unbound drug concentration in the brain and that in plasma at steady state [12]. Summerfield et al. and Langthaler et al. showed, for example, how transport data from MDCKI/II cell lines could be used to predict Kp_uu,brain_ in vivo [13, 14]. Similarly, Uchida et al. and Nicolaï et al. utilized LLC-PK1 cell line data to predict Kp_uu,brain_ in mice, rats, and humans within threefold and twofold error, respectively, compared to observed Kp_uu.brain_ [15, 16]. Measures of Kp_uu,brain_ give important insights into the extent of drug distribution at steady state (SS), but neglect the importance of the rate of distribution and the processes before SS is reached [6, 17]. Since the receptor or target of interest is exposed to fluctuating concentrations of the drug over time after single doses or short infusions, the static, SS-measure of Kp_uu,brain_ is not ideal to predict pharmacodynamic effect(s). In addition, the drug might have to compete with endogenous ligands at its target site, the success of which might change over the course of the treatment based on changing drug concentrations. Understanding and being able to predict a complete, temporal unbound PK profile is therefore crucial to relate drug doses to their ultimate effect.

For prediction of both the rate and extent of distribution, physiologically based pharmacokinetic (PBPK) models can be applied. They allow for predictions of PK profiles by leveraging physiological knowledge, drug-specific properties and mechanistic information from in vitro studies for predictions of drug PK in vivo [18]. Successful development of a generic CNS PBPK model that explicitly takes into account P-gp mediated clearance (CL_Pgp_) at the BBB (and as such predicts both rate and extent of transport) is of significant interest. Such a model would have great potential for clinical applications but also for theoretical investigation, allowing what-if studies that explore P-gp activity specifically (e.g., to explore the impact of disease on P-gp functionality). Some PBPK models that use in vitro data to predict brain PK of P-gp substrates have already been developed [19–26]. Though informative, these models have a number of shortcomings: they (1) commonly rely on the introduction of empirical scaling factors to accurately predict observed data [19, 23, 24], (2) estimate additional transporters aside from P-gp which makes it hard to gauge the success of modelling P-gp [21, 26], and (3) often lay the focus on cerebrospinal fluid (CSF) instead of the more relevant brainECF [19, 24]. Moreover, these models have been validated with a limited number of drugs (no more than three drugs with temporal PK predictions, more often a single drug) [20–26], questioning their general applicability.

A large amount of literature is available on in vitro transport data. It makes sense from a 3R (Reduce, Recycle, Reuse*)* standpoint [27] to repurpose this data for PBPK models instead of setting up a new experimental study. An important factor that has not been investigated sufficiently in the context of CNS PBPK models is the variable nature of in vitro data [28, 29] that can impact the accuracy and robustness of model predictions [30, 31]. Understanding this impact is crucial, especially if an investigation relies on data retrieved from literature as starting point for a computational study. Therefore, it is still unclear to what extent the use of in vitro transport data as input in a CNS-PBPK model allows for *reliable and generic* predictions of the rate and extent of distribution into the brainECF, especially for P-gp substrates. The aim of the current study is to determine whether in vitro data extracted from literature can be repurposed as input in a CNS-PBPK model to confidently predict rat brainECF PK for multiple P-gp substrates. Specifically, we want to establish how variability in transport data from different sources (for the same drug) influences prediction outcomes and model robustness. Importantly, we did not include any empirically derived scaling factors in the methodology, and instead we use a purely bottom-up approach for the prediction of brainECF PK.

## Materials & methods

### Structural overview of the developed CNS PBPK model (LeiCNS-PK3.4)

Predictions of rat brain ECF PK were made by building on the previously published LeiCNS-PK3.0 PBPK model, which is able to accurately predict unbound CNS PK in multiple CNS compartments in rats, mice and humans [32–34]. This model leverages drug physicochemical properties, physiological properties of the CNS, and systemic plasma PK to predict CNS disposition. All changes that we made in the model structure concerned the movement of drugs across the BBB into or out of the brainECF. Though disposition to the cerebral spinal fluid (CSF) is not evaluated in the current study, CSF compartments and distribution are still present in the model structure as previously described [32]. Though P-gp is also present at the blood-CSF-border (BCSFB) [35], this was not taken into consideration since the focus of the current study is on BBB transport.

**Fig. 1:**
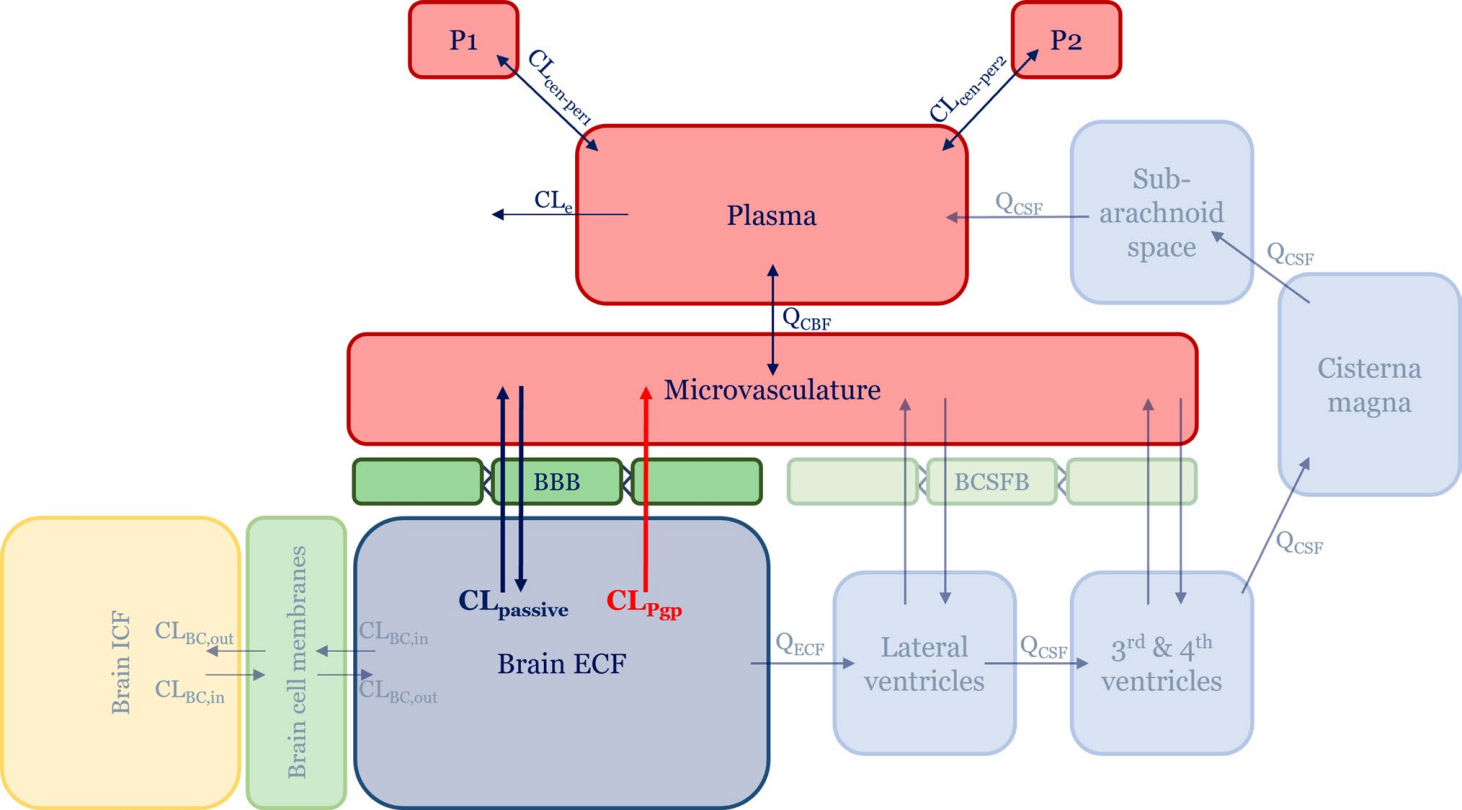
Schematic of the LeiCNS-PK3.4 model structure. The LeiCNS model contains multiple CNS compartments, including intracellular fluid and cerebrospinal fluid compartments. The focus of the current study is on the distribution of drug from the brain microvasculature to the brain extracellular fluid across the blood–brain-border, through the passive clearance (blue bold arrows) and P-gp mediated clearance (CL_Pgp_, red bold arrow). All aspects that are unchanged compared to LeiCNS-PK3.0 and that are not discussed in this study are shown with reduced opacity. *BBB* Blood–brain-border, *BCSFB* Blood-cerebrospinal fluid-border, *CL*_*BC,in*_ Clearance into brain cell membranes, *CL*_*BC,out*_ Clearance out of brain cell membranes, *CL*_*cen-per1/2*_ Intercompartmental clearance, *CL*_*e*_ Systemic clearance, *CL*_*passive*_ Passive clearance, *CL*_*Pgp*_ P-gp mediated clearance, *ECF* Extracellular fluid, *ICF* Intracellular fluid, *P1* Peripheral compartment 1, *P2* Peripheral compartment 2, *Q*_*CBF*_ cerebral blood flow, *Q*_*CSF*_ cerebral spinal fluid flow, *Q*_*ECF*_ ECF bulk flow (Color figure online)

Some changes were made to the LeiCNS-PK3.0 model to obtain the new LeiCNS-PK3.4 model (Fig. 1). First, in the LeiCNS-PK3.0 model, transcellular and paracellular diffusion across the BBB are modelled as separate processes. In our new model these are grouped together into a single CL term for passive diffusion (CL_passive_). This is a step back, but in this case it was needed since the aim of the current study was to evaluate the reliability of in vitro derived data for predicting BBB disposition, and in reported in vitro data there is no distinction between paracellular and transcellular passive transport. Additionally, in this way the LeiCNS-PK3.4 model can be compared directly with previously published PBPK models that apply similar methods for passive diffusion across the BBB [20–23]. Then, as P-gp mediated clearance (CL_Pgp_) is explicitly accounted for in LeiCNS-PK3.4, the asymmetry factor (AF) was not required in the current model (i.e., for P-gp substrates) since the AF accounts for processes that drive Kp_uu,brain_ away from unity, which is mainly driven by P-gp efflux for P-gp substrates. Thus, LeiCNS-PK3.4 can be informed completely by in vitro derived information to describe drug distribution across the BBB.

### Determining drug clearances across the BBB

**Fig. 2:**
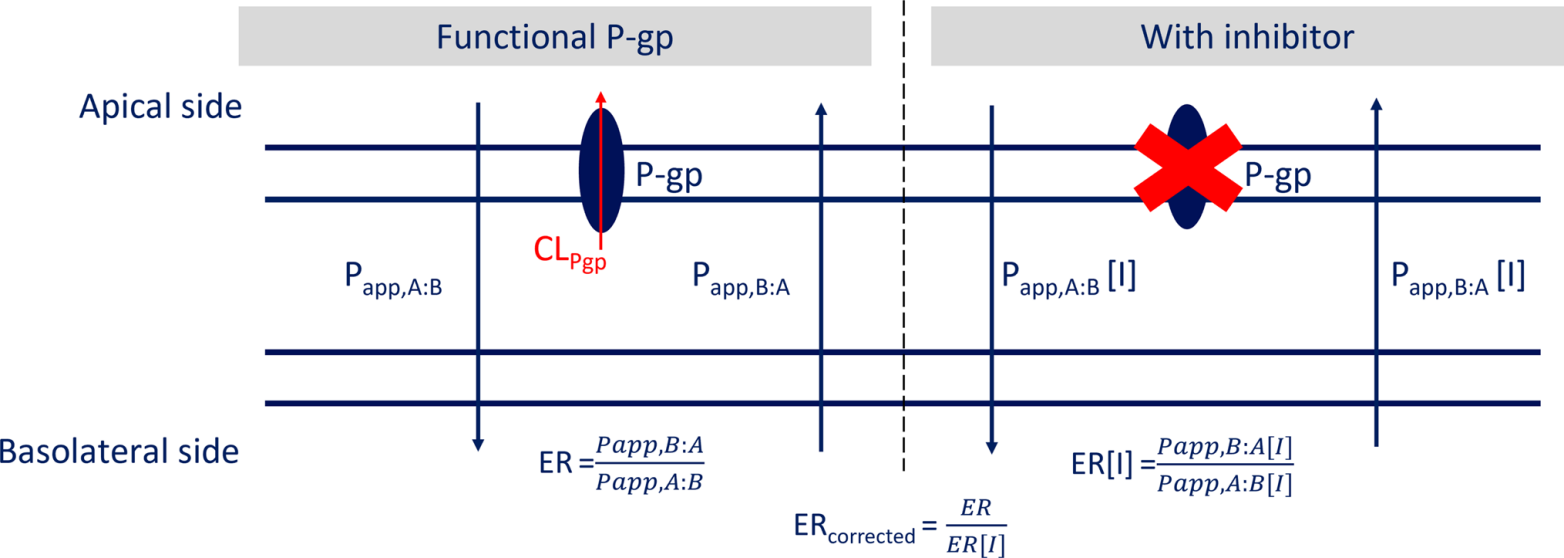
Schematic overview of in vitro transwell assays and related membrane transport parameters. A drug is dosed at either the apical (**A**) or basolateral (**B**) side of a monolayer transfected with P-gp. The rate of appearance in the other chamber is subsequently measured, from which the apparent permeability (P_app_) of a drug from one side of the monolayer to the other can be determined (displayed on the left). Coadministration of an inhibitor, or using a cell line that does not express P-gp, allows for measurements of P_app_ without P-gp (P_app_[I]), which is displayed on the right

All drug movement across the BBB in vivo was predicted by using parameters from transwell permeability assays, as schematically illustrated in Fig. 2. In short, a drug is dosed at either the apical (A) or basolateral (B) side of a monolayer transfected with P-gp. The rate of appearance in the other chamber is subsequently measured, from which the apparent permeability (P_app_) of a drug from each side of the monolayer to the other can be determined [9, 10]. Coadministration of a P-gp inhibitor or using a cell line that is not transfected with P-gp allows for the measurement of permeability without P-gp mediated CL (P_app_[I]). The efflux ratio (ER), which is the ratio of the basolateral to apical (P_app,B:A_) and apical to basolateral (P_app,A:B_) permeability, highlights the degree of efflux mediated by P-gp. To account for the effect of other transporters that might influence the disposition across the membrane, the ER can be divided by the ER obtained when P-gp is inhibited (ER[I]). By doing so, the corrected efflux ratio (ER_c_) is calculated [10, 36], which represents the effect that solely P-gp has on asymmetric transport across the monolayer (see Fig. 2).

Passive diffusion of drugs across the BBB in vivo (CL_passive_, Fig. 1) was calculated as [20–23]:

1 $$C{L}_{passive}= {P}_{app,A:B}\left[I\right]*S{A}_{BBB}*60$$

where P_app,A:B_[I] is the apparent permeability (× 10^–6^ cm/s) from the apical to the basolateral direction with inhibition of P-gp (or derived from a cell line not transfected with P-gp) and SA_BBB_ is the surface area of the BBB in rats (155 cm^2^ [32]). The expression was multiplied by 60 to express CL_passive_ in mL/min. The P-gp mediated clearance (CL_Pgp_) was determined through the method adapted from Kalvass et al. [37]:

2 $${CL}_{Pgp}= {(ER}_{c}-1)*2{P}_{app,A:B}\left[I\right]*S{A}_{BBB,trans}*REF* 60$$

where ER_c_ is the corrected efflux ratio in vitro (see Fig. 2) and SA_BBB,trans_ is the surface area of the rat BBB available for transcellular processes (SA_BBB,trans_ = 0.998 * SA_BBB_ = 154.69 cm^2^ [32]), since P-gp is embedded in the apical membrane of endothelial cells [38]. The multiplication by 60 is done to express CL_Pgp_ in mL/min.

Additionally, to account for differences in expression and activity of P-gp between in vitro and in vivo, we applied a proteomics-informed relative expression factor (REF) [15, 20, 39]:

3 $$REF= \frac{Pgp \, expression \, in \, vivo}{Pgp\, expression\, in\, vitro}$$

where the P-gp protein expression in vivo and in vitro is given in fmol/µg total protein. Of note is that use of the REF assumes the existence of a linear correlation between expression of P-gp and its activity [40, 41], and that its intrinsic activity (i.e., the activity of one entity of P-gp) is system-independent.

Equations (1) and (2) were subsequently introduced into LeiCNS-PK3.4. Changes in the amount of drug in the model compartments over time were described through ordinary differential equations. A change in unbound drug in the brainECF compartment of the final model was defined as:

4 $${V}_{ECF}\frac{d{C}_{ECF}}{dt}=C{L}_{passive}*{f}_{up}*{C}_{MV}-\left({CL}_{Pgp}+C{L}_{passive}\right)*{C}_{ECF}-{Q}_{ECF}*{C}_{ECF}- {CL}_{BC,in}* {C}_{ECF}*PH{F}_{ECF}+{CL}_{BC,out}*{C}_{BC}$$

where V_ECF_ is the volume of the brainECF and C_MV_, C_ECF,_ and C_BC_ are drug concentrations in the brain microvasculature, brainECF, and brain cell membranes, respectively. *f*_*up*_ is the unbound fraction of drug in plasma, and Q_ECF_ is the bulk flow of brainECF to the lateral ventricles. CL_BC,in_ and CL_BC,out_ are clearances that describe drug partitioning into and out of brain cell membranes, and were calculated as described previously [32]. PHF_ECF_ is the fraction of drug that is unionized and available for partitioning into the brain cell membranes from the ECF [32]. All concentrations and clearances are expressed in ng/mL and mL/min, respectively.

### In vivo microdialysis data for model evaluation

The developed model was evaluated using in vivo unbound brainECF PK profiles obtained through microdialysis in rats. Predictions of passive diffusion across the BBB were evaluated using the plasma and brainECF PK data on the passively diffusing drugs acetaminophen, raclopride, and paliperidone (which is a P-gp substrate) co-administrated with tariquidar (a P-gp inhibitor). All in vivo data for the passively diffusing drugs were published previously and available in-house [42]. The prediction of P-gp functionality was evaluated with the P-gp substrates morphine, quinidine, risperidone, paliperidone (without tariquidar co-administration), and verapamil. A distinction was made between microdialysis data obtained from (ultra) short infusion of the drug (intravenous [I.V.] and subcutaneous [S.C.]) and data from a loading dose and maintenance constant rate infusion. The short infusion in vivo data were reported previously and available in-house for morphine [43], quinidine [44], and paliperidone [45]. For risperidone, data were extracted from literature [46]. The risperidone brainECF data were corrected for microdialysis lag time. Morphine plasma PK data concerned total plasma concentrations and were corrected for plasma protein binding in the model structure (*f*_*up*_ in Eq. (4)). Continuous infusion data were available for paliperidone, quinidine, risperidone and verapamil and were extracted from the study by Nagaya et al. [47]. An overview of the different drugs, doses, and references are shown in Table 1.

**Table 1:** Summary of the dosing regimens

	Drug	Administration route	Total dose (mg/kg body weight^*^)	Infusion time (minutes)	References
Short infusion	Acetaminophen	I.V	15	10	[48]
	Raclopride	I.V	0.56	10	[42]
	Morphine	I.V	40	10	[43]
	Paliperidone	I.V	0.5	20	[45]
	Quinidine	I.V	20	10	[44]
	Risperidone	S.C	3	N/A	[46]
Continuous infusion	Paliperidone	I.V	0.8 (L)/4 (M)	240	[47]
	Quinidine	I.V	8.0 (L)/20 (M)	240	[47]
	Risperidone	I.V	0.7 (L)/4 (M)	240	[47]
	Verapamil	I.V	0.9 (L)/4 (M)	240	[47]

*I.V* Intravenous, *S.C* subcutaneous, *N/A* not available for the dosage form, *(M)* maintenance dose, *(L)* loading dose. *Body weight of a typical rat was set to 0.25 kg in the predictions

### Overview of input parameters

Input parameters for the CNS PBPK model can be divided into four types: drug physicochemical parameters, physiological parameters, plasma pharmacokinetic parameters and drug transport parameters. Drug physicochemical properties and rat CNS physiological properties are listed in the Supplementary Tables 1 and 2. The plasma PK profile of a drug served as an input function for the model to predict distribution into the CNS and is described with an empirical population PK model. All PK models for the short infusion data were reported previously [32, 42, 46]. The risperidone PK parameters were adapted so that all parameters are expressed in millilitres and describe a typical rat weighing 0.25 kg. The continuous infusion plasma PK data were not described well using the previously reported PK models. As this is a prerequisite to evaluate the accuracy of the P-gp mediated CL, the PK models were re-estimated to fit a one compartment PK model using Monolix (version 2023R1, Lixoft, Antony, France) [49]. The PK parameters used to describe systemic plasma concentrations of the drugs are shown in Table 2.

**Table 2:** Pharmacokinetic parameters of the validation drugs in rats

	Drug	CL_cen_ (mL/min)	Q_cen-per1_ (mL/min)	Q_cen-per2_ (mL/min)	V_cen_ (mL)	V_per1_ (mL)	V_per2_ (mL)	K_a_ (1/min)
Short infusion	Acetaminophen (1)	4.7	11.2	31.4	50.7	27892	162.5	N/A
	Raclopride (1)	47.7	16.5	56.8	83	603	457.5	N/A
	Morphine (2)	22.6	30.8	7.2	152	530	1200	N/A
	Paliperidone (1)	219.5	6766	0	25	32,981	0	N/A
	Quinidine (1)	178.3	238	754	184	7335	5063	N/A
	Risperidone (3)	77	0	0	5250	0	0	0.03
Continuous infusion	Paliperidone (4)	29.2	0	0	2053	0	0	N/A
	Quinidine (4)	25	0	0	7314	0	0	N/A
	Risperidone (4)	37	0	0	3799	0	0	N/A
	Verapamil (4)	72.3	0	0	11,700	0	0	N/A

(1): From Saleh et al. (2021) [32]. (2) From Yamamoto et al (2017)^42^. (3): From Cremers et al. (2012) [46], recalculated for a rat weighing 0.25 kg. (4): Estimated

The transport parameters of each drug (i.e., P_app,A:B_, P_app,B:A_, P_app,A:B_[I], P_app,B:A_[I], ER, and ER_c_), and P-gp expression in vitro and in vivo were retrieved from previously published literature (Table 3). Values were retrieved from multiple sources using multiple cell lines, where possible, to assess the influence of different experimental conditions and variability in reported parameter values on model functionality.

**Table 3:** Apparent passive permeability P_app,A:B_[I] and corrected efflux ratio (ER_c_) of P-gp substrates derived from literature

Drug	Cell line	P_app,A:B_[I] (× 10^–6^ cm/s)	ER_c_	References
Acetaminophen	Caco-2	31.9*	1	Kamiya et al. (2020) [50]
Raclopride	Caco-2	73.4 [Zosquidar, KO-143, Benzbromarone]	1.1†	Colclough et al. (2024) [51]
Morphine	Caco-2	2.08 [Cyclosporine A]	1.6	Crowe (2002)[52]
	MDCKII-MDR1	2.12	1.3	Verscheijden et al. (2021) [20]
	MDCKII-MDR1	1.8	1.3	Feng et al. (2008) [53]
	MDCKII-MDR1	4.8	1.9	Garberg et al. (2005) [54]
Paliperidone	LLC-PK1-mdr1a	14.7	8.1	Inoue et al. (2012) [55]
	LLC-PK1-MDR1	13.3	5.3	Inoue et al. (2012) [55]
	MDCKII-MDR1	16.8	3.3	Feng et al. (2008) [53]
Quinidine	Caco-2	52.0 [GF120918]	4.3	Korjamo et al. (2006) [56]
	Caco-2	58.9 [Verapamil]	1.5	Mukkavilli et al. (2017) [57]
	Caco-2	54.5 [GW918]	5.2	Troutman & Thakker (2003a) [58]
	LLC-PK1-mdr1a	56.90	16.1	Nicolaï et al. (2020) [16]
	LLC-PK1-mdr1a	57.2	32.8	Uchida et al. (2011) [15]
	LLC-PK1-MDR1	15.9	12.9	Nagaya et al. (2020) [59]
	MDCKII-MDR1	9.52	5.9	Bicker et al. (2017) [60]
	MDCKII-MDR1	8.0	7.4	Feng et al. (2008) [53]
	MDCKII-MDR1	36.8	7.3	Troutman & Thakker (2003b) [61]
Risperidone	Caco-2	1.56 [Verapamil]	4.1	Cousein et al. (2007) [62]
	LLC-PK1-mdr1a	15.8	3.6	Inoue et al. (2012) [55]
	LLC-PK1-mdr1a	66.2	2.1	Nicolaï et al. (2020) [16]
	LLC-PK1-mdr1a	96.3	10.6	Uchida et al. (2011) [15]
	LLC-PK1-MDR1	14.0	3.4	Inoue et al. (2012) [55]
	LLC-PK1-MDR1	15.6	3.9	Nagaya et al. (2014) [63]
	MDCKII-MDR1	19.8	2.0	Feng et al. (2008) [53]
	MDCKII-MDR1	53.6 [GF120918]	1.7	Mahar Doan et al. (2002) [9]
Verapamil	LLC-PK1-mdr1a	41.5	2.9	Nicolaï et al. (2020) [16]
	LLC-PK1-mdr1a	73.5	13.3	Uchida et al. (2011) [15]
	LLC-PK1-MDR1	22.3	5.4	Nagaya et al. (2014) [63]
	LLC-PK1-MDR1	47.1	2.7	Nicolaï et al. (2020) [16]
	MDCKII-MDR1	12.6	2.1	Feng et al. (2008) [53]
	MDCKII-MDR1	44.0 [GF120918]	1.74	Mahar Doan et al. (2002) [9]
	MDCKII-MDR1	28.6	2.65	Troutman & Thakker (2003b) [61]
	MDCKII-MDR1	30.0	3.10	Garberg et al. (2005) [54]

For P_app,A:B_[I], the P-gp inhibitor used to measure passive diffusion is given in square brackets. When no inhibitor is specified, P_app,A:B_[I] was determined in parental cell lines. *Assumed P_app,A:B_[I] to be the same as P_app,A:B_ without inhibitor due to not being transported by P-gp. † Efflux ratio determined in MDCKI-MDR1 cell line

### Model evaluation

Model evaluation was done by calculating prediction errors (PEs) [20, 45] as:

$$PE= \frac{{Y}_{pred,j}-{Y}_{obs,i,j}}{({Y}_{pred,j}+{Y}_{obs,i,j})/2}$$

where Y_pred,j_ is the model predicted typical concentration at time point *j* and Y_obs,i,j_ is the observed concentration for the *ith* individual at time point *j*. An optimal prediction would render a median PE of 0, but predictions were deemed accurate when the median PE fell within twofold error (−0.67 ≤ PE ≤ 0.67) compared to the observed data.

### Software

Plasma PK parameters for the continuous infusion datasets were estimated using Monolix (version 2023R1, Lixoft, Antony, France) [49]. Literature data reported in plots was extracted using WebPlotDigitizer version 4.6 [64]. Development, execution of the LeiCNS-PK3.4 model and subsequent visualizations of the results were all done using RStudio version 4.3.0 [65]. The model was simulated using the freely available R package rxode2 version 2.0.13, while data visualization was done using the R package ggplot2.

## Results

### Reported apparent permeability of P-gp substrates in literature

In total, 34 sets of in vitro passive permeability (P_app,A:B_[I]) and corrected efflux ratio (ER_c_) for the drugs were retrieved from previously published literature. Cell lines used for the transport assays were Caco-2, MDCKII transfected with human P-gp (MDR1), and LLC-PK1 transfected with either rodent (mdr1a) or human (MDR1) P-gp. For the P-gp substrates, P_app_ values were available for quinidine and risperidone from all cell lines, while information on morphine, paliperidone and verapamil permeability was missing for either LLC-PK1 (morphine) or Caco-2 (paliperidone and verapamil).

**Fig. 3:**
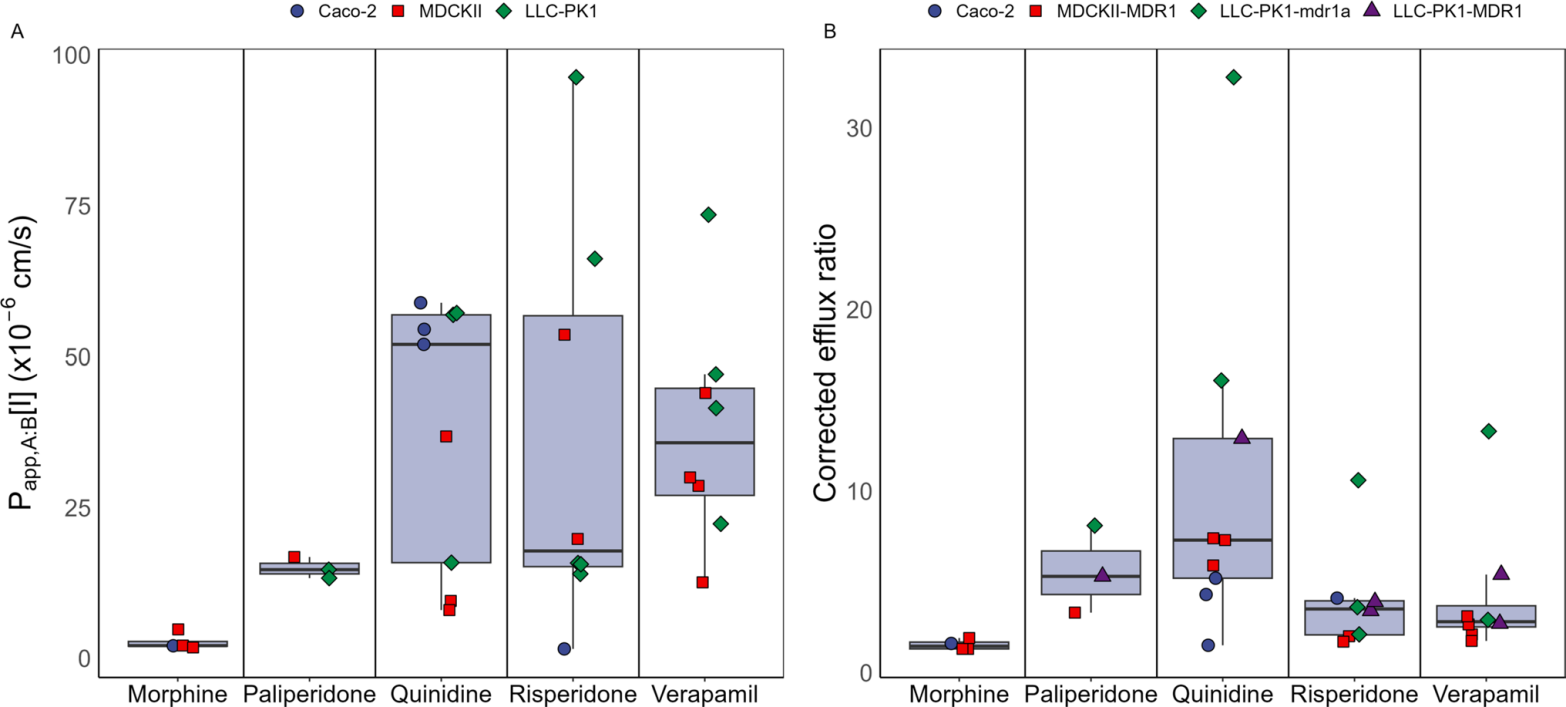
Overview of **A** P_app,A:B_[I] and **B** corrected efflux ratio (ER_c_) per drug. Different cell lines are indicated by shapes and colours of the observed datapoints (Color figure online)

In vitro transport values extracted from literature showed varying degrees of variability of P_app,A:B_[I] and ER_c_ for the P-gp substrates (Fig. 3 and Table 3). Similar P_app,A:B_[I] and ER_c_ between different sources and cell lines was observed for morphine (mean P_app,A:B_[I] ± SD = 2.7 ± 1.2 × 10^–6^ cm/s, mean ER_c_ ± SD = 1.5 ± 0.2) and paliperidone (mean P_app,A:B_[I] ± SD = 14.9 ± 1.4 × 10^–6^ cm/s, mean ER_c_ ± SD = 5.6 ± 2.0). However, the P_app,A:B_[I] of quinidine, risperidone and verapamil showed a higher degree of variability, with risperidone passive permeability ranging approximately 62-fold between sources. This variation was not distinctly related to a specific cell line, and large variability could be observed within a single cell line. For example, risperidone P_app,A:B_[I] values measured in LLC-PK1 cells ranged from 15.8 × 10^–6^ to 96.3 × 10^–6^ cm/s (mean ± SD of 41.6 ± 33.8 × 10^–6^ cm/s). For quinidine, P_app,A:B_[I] was similar for Caco-2 and LLC-PK1, except for the value reported by Nagaya et al. in LLC-PK1 cells (mean ± SD of 55.9 ± 2.4 × 10^–6^ cm/s without Nagaya et al. P_app,A:B_[I]). Permeability of quinidine in MDCKII cells was lower than in Caco-2 and LLC-PK1 and also varied, with the values reported by Bicker et al. and Feng et al. aligning with a mean of 8.76 × 10^–6^ cm/s, whereas the P_app,A:B_[I] from Troutman et al. was higher (36.8 × 10^–6^ cm/s).

Though the risperidone and verapamil P_app,A:B_[I] values vary substantially, the ER_c_ values were more consistent between sources, except for the ER_c_ reported by Uchida et al. which were higher than the other values for both drugs. Like the P_app,A:B_[I], the quinidine ER_c_ showed great variability (mean ER_c_ ± SD = 10.4 ± 9.0), with the reported ER_c_ varying up to 22-fold between Caco-2 and LLC-PK1-mdr1a based studies.

### P-gp expression is constant in vivo but variable in vitro

In vivo P-gp expression in rat brain microvascular endothelial cells was constant between different sources, with a mean of 19.4 fmol/µg total protein (see Supplementary Table 3). In contrast, in vitro expression of P-gp in the cell lines showed a high degree of variability (see Supplementary Table 3). Table 4 shows the highest and lowest reported P-gp protein expression in each cell line, as well as the average of these values. Only one P-gp expression level was reported for the LLC-PK1-MDR1 cell line, and this was categorized under the lowest expression due to its proximity to the lowest value of the LLC-PK1-mdr1a cell line. This is however an arbitrary distinction.

**Table 4:** Expression of P-gp in different cell lines in vitro

Cell line	Highest P-gp expression (fmol/µg protein)	Average P-gp expression (fmol/µg protein)*	Lowest P-gp expression (fmol/µg protein)
Caco-2	7.4^$^	4.7	2
LLC-PK1-MDR1	–	–	13.1
LLC-PK1-mdr1a	61	38.1	15.2
MDCKII-MDR1	10.3	6.3	2.2^#^

Due to variability in the reported values, the expression values are categorized into the highest, lowest, and average reported expression levels. All P-gp expression levels and references are given in Supplementary Table 3 *Average of highest and lowest expression values. ^$^Mean of the P-gp expressions reported by Harwood et al. (2016) [66] and Miliotis et al. (2011) [67]. ^#^Mean of the P-gp expressions reported by Di et al. (2011) [68], Feng et al. (2019) [69], and Jacqueroux et al. (2020) [70]

### BrainECF PK is predicted well for passively diffusing drugs

**Fig. 4:**
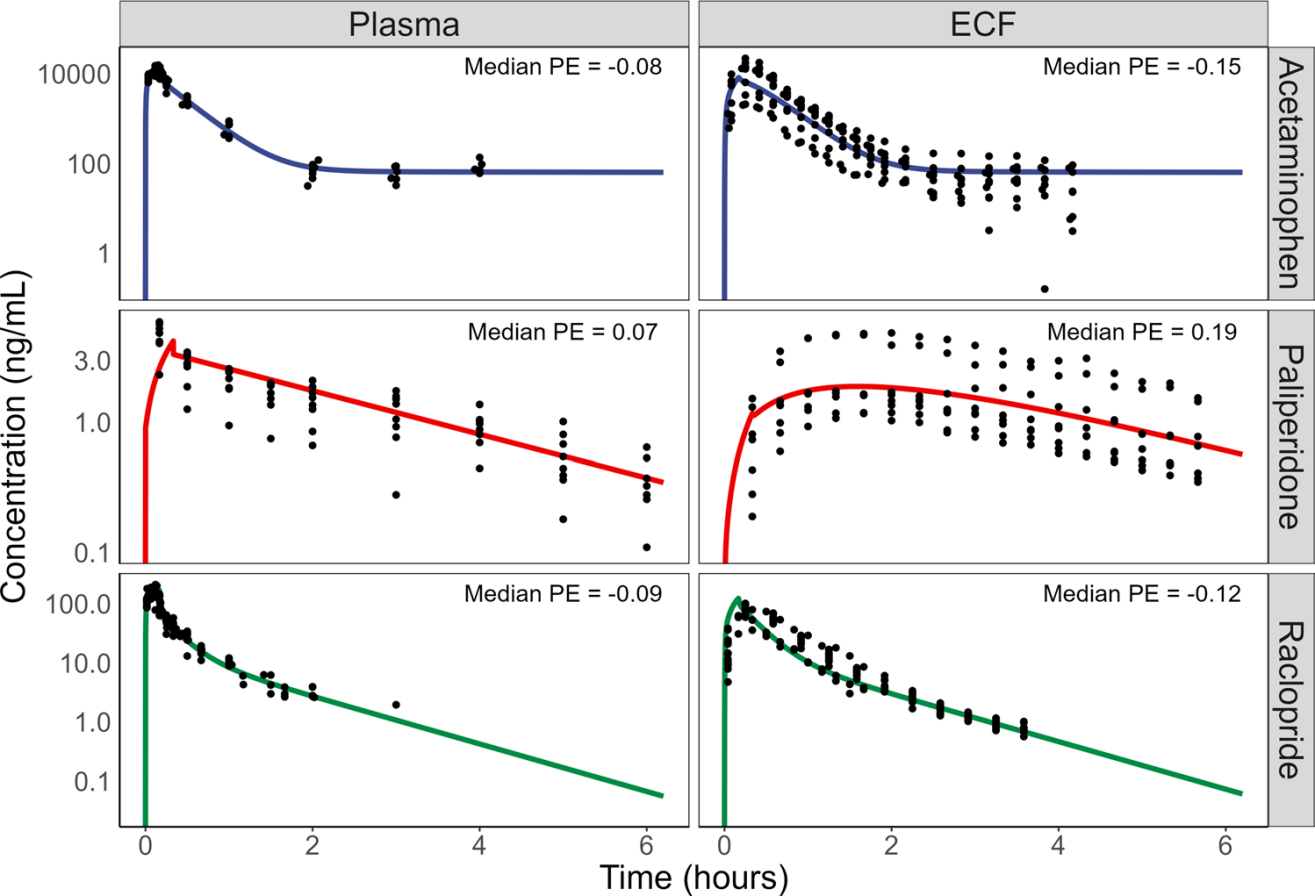
Description of unbound plasma PK and prediction of rat brainECF PK of the passively diffusing drugs acetaminophen, paliperidone co-administered with the P-gp inhibitor tariquidar, and raclopride. These predictions serve as a validation of whether the model is able to accurately predict brain distribution in the absence of P-gp mediated transport. Median PE (calculated as outlined in methods Sect. “Model evaluation”) is shown in the top-right of each subfigure. For the paliperidone prediction, the P_app,A:B_[I] value of 14.7 * 10^–6^ cm/s from Inoue et al. was used

Predictions of brainECF PK were first evaluated for drugs undergoing passive diffusion, to ensure adequate model functionality before including CL_Pgp_. BrainECF PK was predicted for three drugs that diffuse passively across the BBB, namely acetaminophen, raclopride and, in case of P-gp inhibition by tariquidar, paliperidone (Fig. 4). Plasma PK data of these drugs were described well by the plasma PK model, and the LeiCNS-PK3.4 model adequately predicted the observed brainECF PK profiles within a two-fold median prediction error (PE). The raclopride prediction did slightly underestimate the time to maximum concentration (T_max_) observed in the brainECF.

### Brain ECF PK prediction accuracy varies between P-gp substrates and dosing schemes

**Fig. 5:**
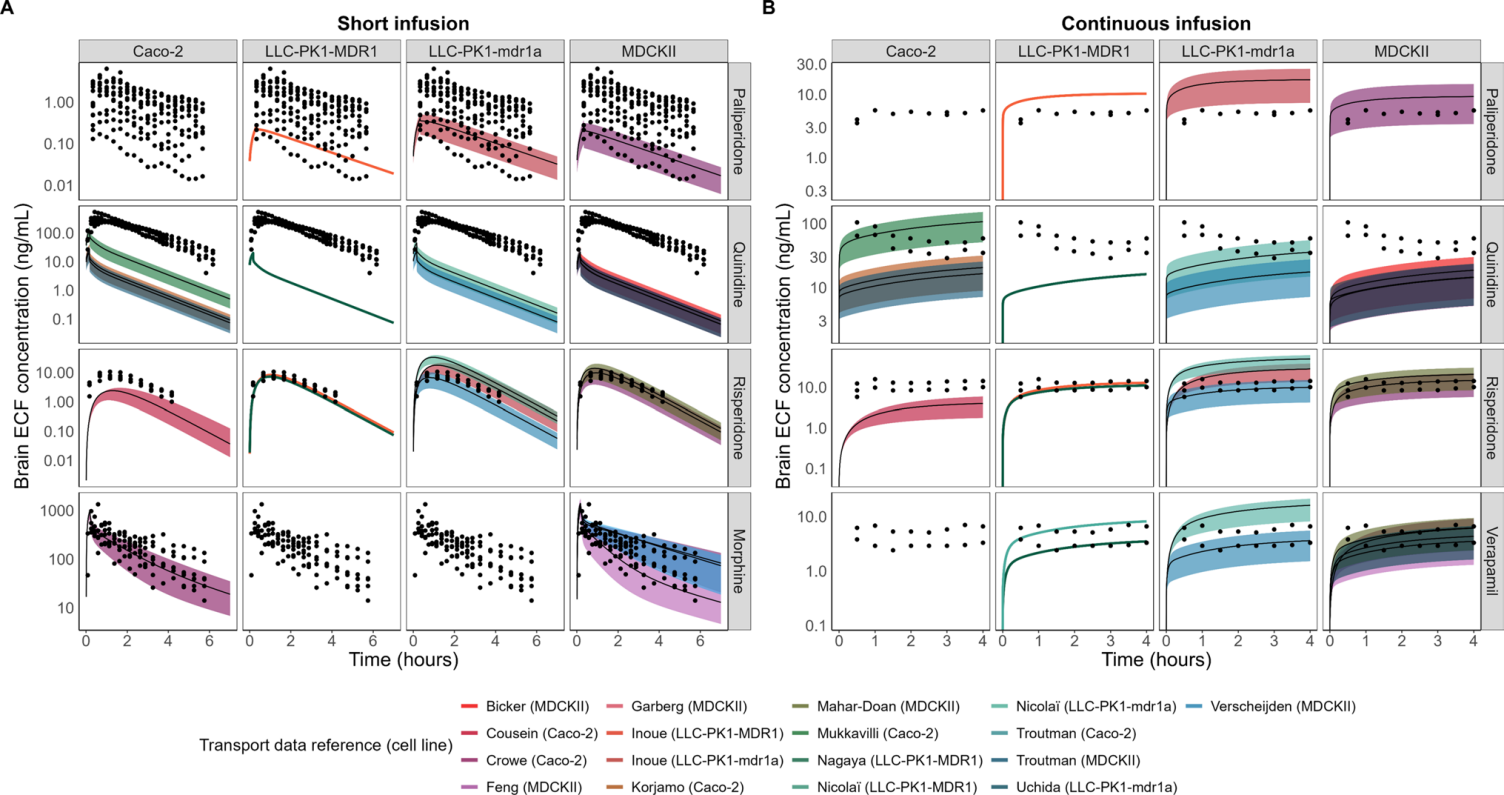
Rat brainECF PK predictions after **A** short infusion (or subcutaneous dose administration) and **B** continuous infusion after loading dose of the P-gp substrates paliperidone, quinidine, risperidone, morphine and verapamil. Each column shows which cell line was used to determine P_app_[I] and ER_c_ values that were used as input for the prediction. The colour of the prediction interval corresponds to the colours given below the table, which specifies the reference from which transport data were retrieved (shown in Table 3). The upper line of the prediction bands indicates the predictions made using the highest in vitro P-gp expression value for the given cell line, while the lowest line of the band corresponds to the lowest expression value. Black lines indicate the prediction made using the average in vitro P-gp expression. Each row indicates a different drug. Plots without predictions indicate lack of transport data in a cell line for a given drug. Observed unbound brainECF concentrations are shown as black points

Predictions of rat brainECF PK were made for five P-gp substrates (Fig. 5). Observed plasma PK data (Supplementary Fig. 1) were described accurately within twofold PE. To account for the reported variability in P-gp expression in vitro, predictions of P-gp substrates’ brainECF PK were made using the lowest, average, and highest P-gp expression levels reported for each cell line (Table 4). The resulting brainECF predictions are represented through a prediction interval, with the upper and lower bounds of the coloured bands in Fig. 5 corresponding to the predictions made with the highest and lowest in vitro P-gp expression, respectively. The predictions for the average in vitro P-gp expression are shown as a black line. As such, each transport value has three predictions associated to it, except for the LLC-PK1-MDR1 based predictions, since only one in vitro expression value was reported for this cell line.

After administration of a short infusion (or subcutaneous dose), the morphine and risperidone brainECF predictions were accurate across the different transport values and expression levels (Fig. 5A). An overview of the percentage of the predictions that fall within twofold prediction error (PE) per in vitro expression level is shown in Table 5. The PEs for every single prediction (i.e., predictions of each individual reference and per expression level) are shown in Supplementary Tables 4 and 5. Table 5 also shows the accuracy of predictions that were made without the REF (predictions shown in Supplementary Fig. 2). All the morphine brainECF predictions fell within twofold median PE using the average in vitro P-gp expression value (Table 5). The risperidone brainECF predictions showed the best performance with the average or low P-gp expression, with only one of the references being unable to accurately predict the observed brainECF data with any of the P-gp expression values (Cousein et al., using Caco-2 cells, supplementary Table 4). None of the paliperidone or quinidine short infusion predictions fell within twofold PE (Table 5), with the quinidine predictions reaching ± 100-fold underprediction (Supplementary Table 4).

**Table 5:**
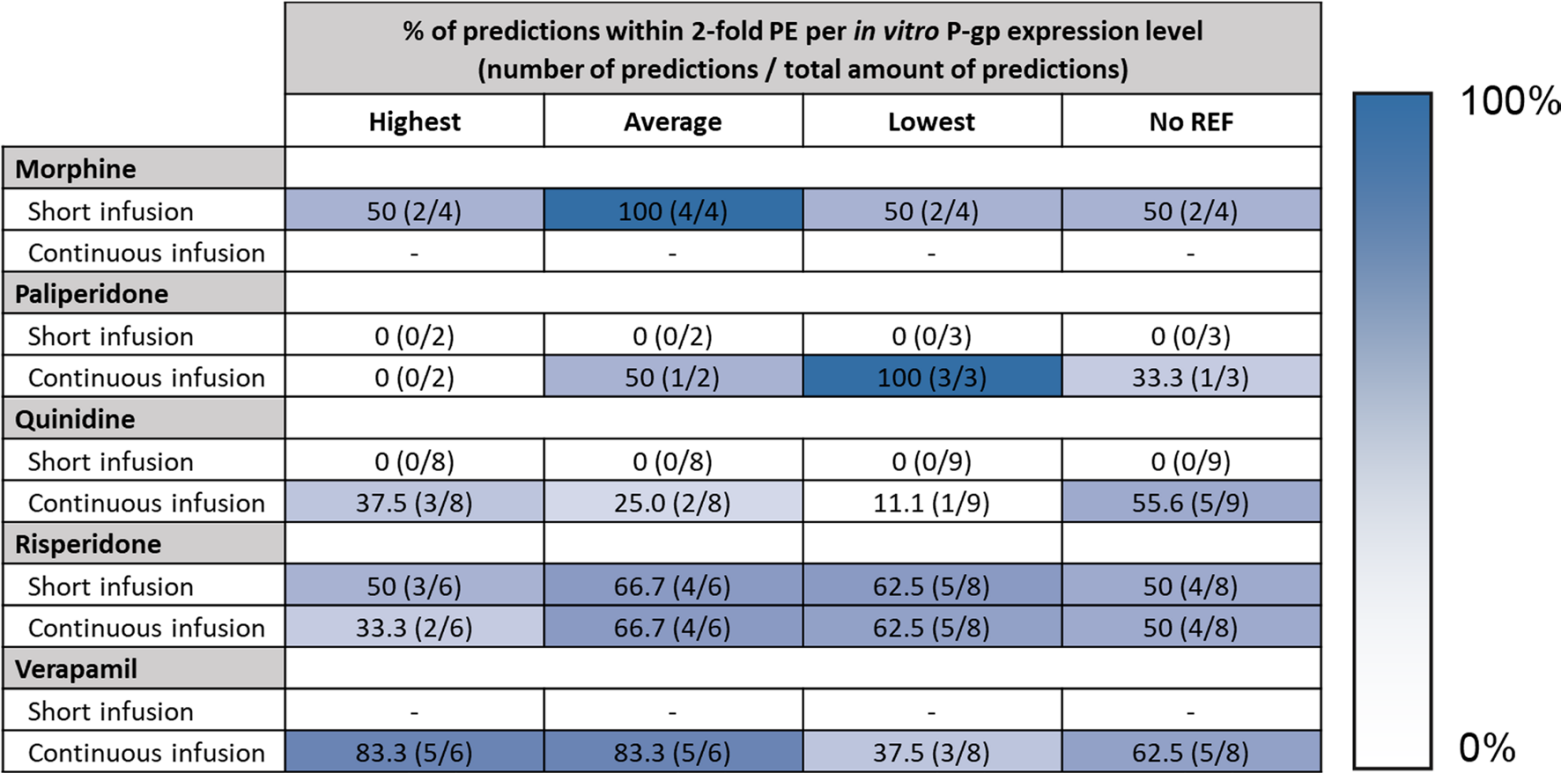
Percentage of all predictions that fall within twofold median prediction error (PE) for each drug per in vitro expression value used to inform the REF

The prediction accuracy for each drug for both the short infusion data (Fig. 5A) and the continuous infusion data (Fig. 5B) is shown. Aside from the in vitro expression levels, the model accuracy when not using a REF is shown (“No REF” column). The number of predictions that are successful compared to the total amount of predictions for a given drug at a certain in vitro expression level are given in brackets behind the percentage. Darker blue cells indicate that more predictions fall within twofold PE, while lighter cells correspond to fewer successful predictions

For the continuous infusion data (Fig. 5B), the observed verapamil brainECF PK was predicted well, with almost all the predictions falling within twofold median PE using either the highest or average in vitro P-gp expression. The brainECF PK predictions for a continuous infusion of risperidone showed similar trends as observed for the predictions after subcutaneous administration (compare Fig. 5A and B). The paliperidone brainECF predictions during the continuous infusion tend to slightly overpredict the observed data but showed < twofold PE with the low in vitro expression value in each cell line (Table 5). Overall, the continuous infusion quinidine data tended to be underpredicted, with 37.5% of the predictions falling within twofold using the high P-gp expression.

### Assessment of prediction accuracy based on different stratifications of the input

**Fig. 6:**
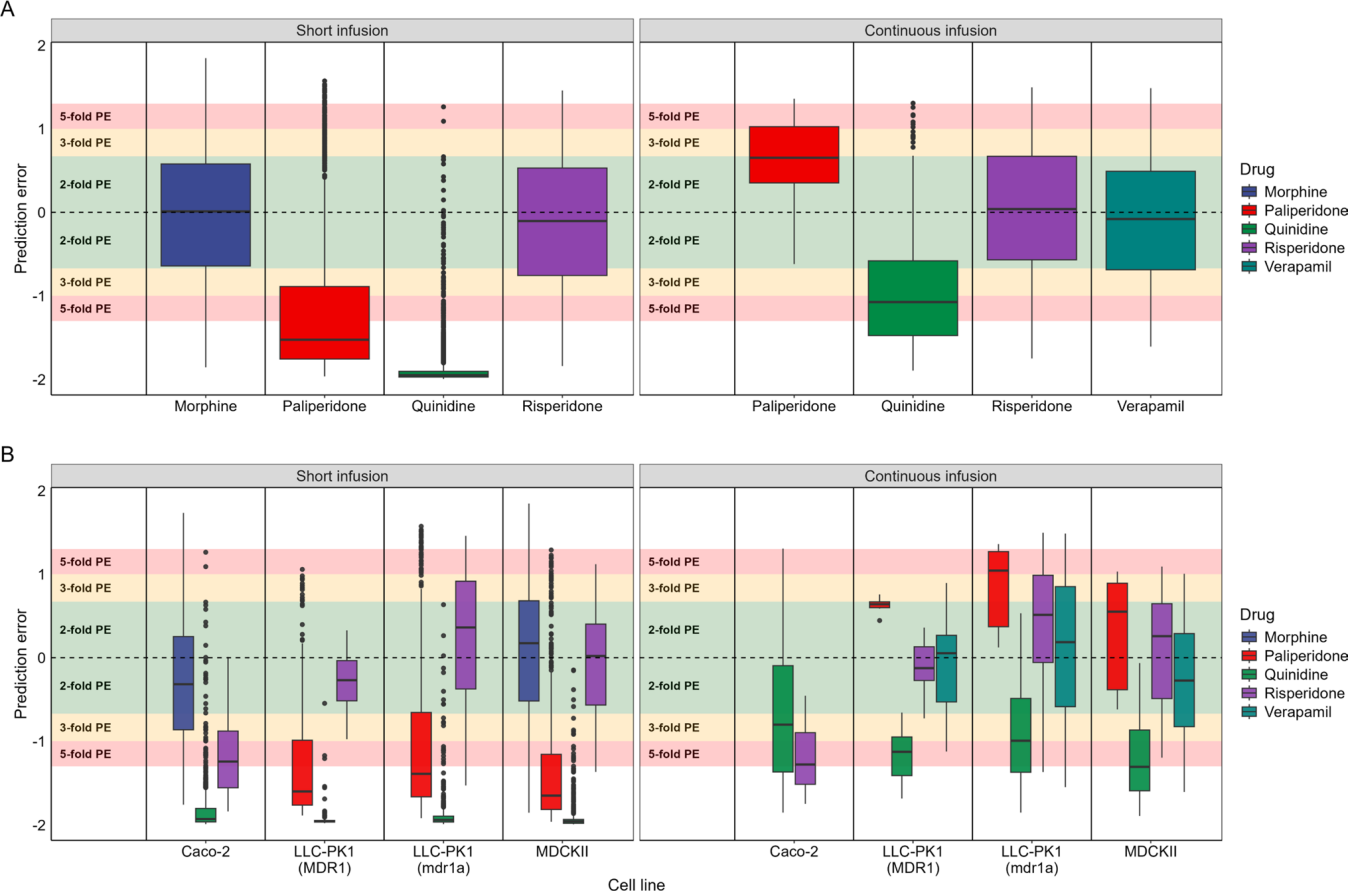
Prediction errors (PE) of the short infusion (left panel) and the continuous infusion with loading dose (right panel) datasets. **A** Overall prediction errors per drug by considering all of the predictions regardless of cell line or in vitro expression level used as input. **B** Prediction errors of the model per drug stratified by the in vitro cell lines. All expression levels (highest, average, and lowest) are considered. Colours of the boxes correspond to the drug in both subplots (**A**) and (**B**). The areas corresponding to < 2-, < 3- and < fivefold over- and underprediction are highlighted in green, yellow and red, respectively. The PE of 0 is indicated by a dashed line. Black dots show outliers that fall outside of the minimum or maximum boxplot range (Color figure online)

Next, we assessed the accuracy of the model predictions when stratifying on different factors. Taking all predictions together (i.e., regardless of cell line or in vitro expression level), the model accurately predicted brainECF PK profiles within twofold median PE for 2 of the 4 short infusion drug dosing regimens and 3 of the 4 drugs for the continuous infusions (Fig. 6A). Across all cell lines, the LLC-PK1-MDR1, LLC-PK1-mdr1a and MDCKII cell lines showed similar prediction accuracies for the different drugs (Fig. 6B). The MDCKII cell lines however showed a higher degree of overlap between predictions than the LLC-PK1-mdr1a cells (Fig. 5).

**Fig. 7:**
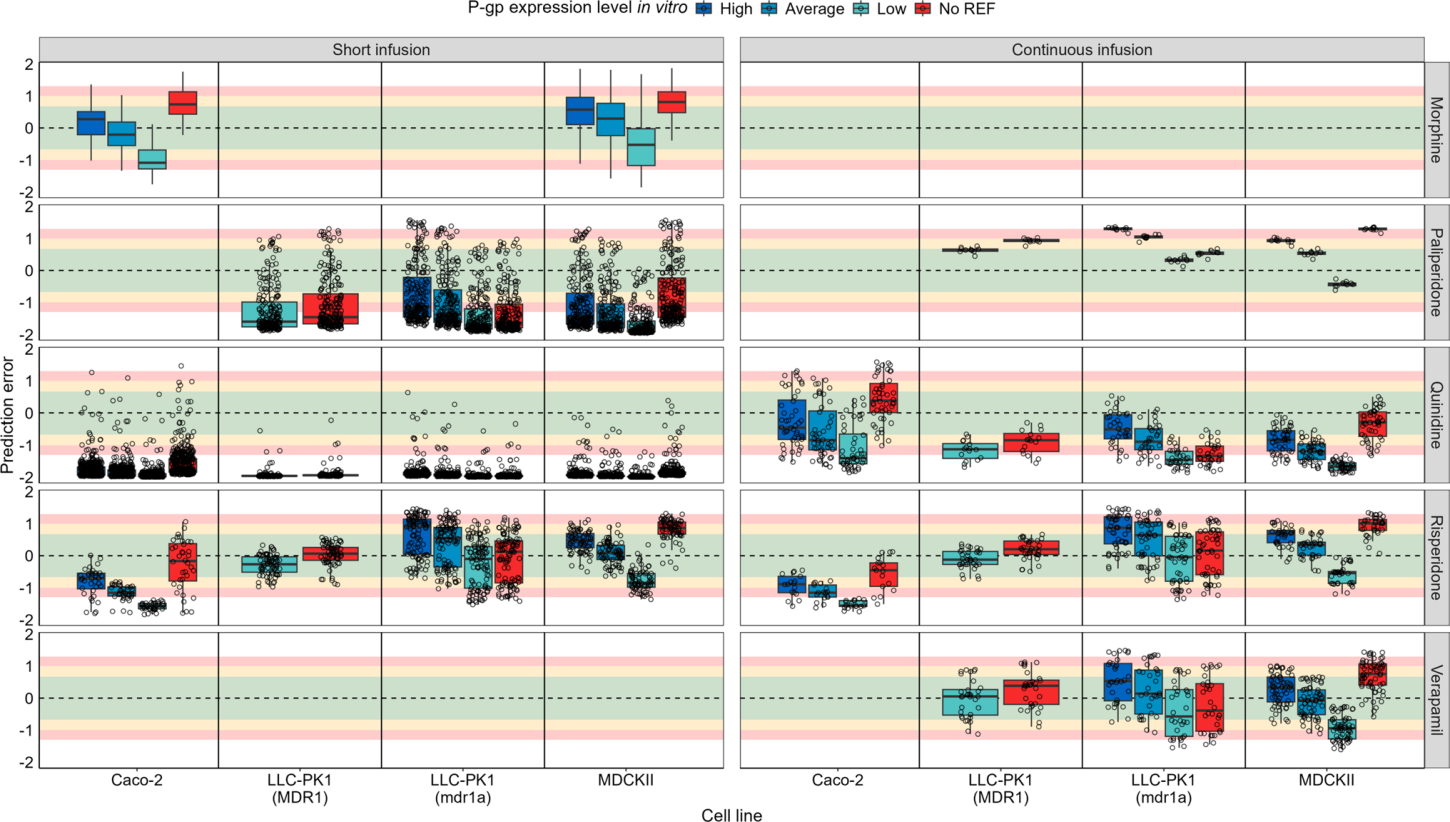
Prediction errors of the model brainECF PK predictions for each drug (rows), stratified by the cell line used in-vitro to determine transport parameters (columns) and the in-vitro P-gp expression used for the scaling of CL_Pgp_ from in vitro to in vivo (box colour). In addition to the predictions made using the three in vitro expression values, the predictions made without considering the REF are shown (red boxplots). The areas corresponding to < 2-, < 3- and < fivefold over- or underprediction are highlighted in green, yellow, and red, respectively. The PE of 0 is indicated with a dashed line. To enhance the clarity of the figure, individual points for morphine were omitted (Color figure online)

Lastly, the brainECF prediction accuracies were investigated per cell line and expression level for each drug (Fig. 7). It shows that there is not one clear in vitro expression value that is the most accurate for predictions, which can also be concluded from the results in Table 5. Instead, to what extent P-gp activity has to be modulated appears to be drug dependent, as can for example be seen for the continuous infusion predictions obtained using data from the LLC-PK1-mdr1a cell line as input. Here, paliperidone and risperidone required the lowest in vitro expression value, verapamil the average value, and quinidine the highest in vitro expression value. In theory, the REF value used for scaling should depend on the experimental condition only and not on the drug. A few studies reported transport values for multiple drugs (i.e., values were obtained under the same experimental condition), and plotting the prediction errors of the continuous infusion data per drug showed that a single scaling factor (e.g., low expression) results in different prediction accuracies between the drugs, even when transport values were derived from the same experimental setup (Supplementary Fig. 3).

### Using in vitro expression and permeability data from the same experiment does not guarantee an accurate brainECF prediction

The risperidone, quinidine and verapamil permeability data extracted from Uchida et al. and Nicolaï et al. were reported in conjunction with the expression of P-gp in the studied cell line (lowest and highest expression in LLC-PK1-mdr1a cells, respectively). Combining the expression and transport data from the same experimental system should, in theory, give the best predictions. Whenever the transport values and P-gp expression data were correctly matched, almost all predictions were more than three-fold over or underpredicted, whereas using the incorrect in vitro expression value (i.e., the expression reported by the other reference) gave better (within twofold PE) predictions (Table 6). For the LLC-PK1-MDR1 based prediction of verapamil, matching the transport values reported by Nicolaï et al. to the P-gp expression reported for LLC-PK1-MDR1 cells in the same article (13.1 fmol/µg) did give an accurate prediction (median PE = 0.28).

**Table 6:**
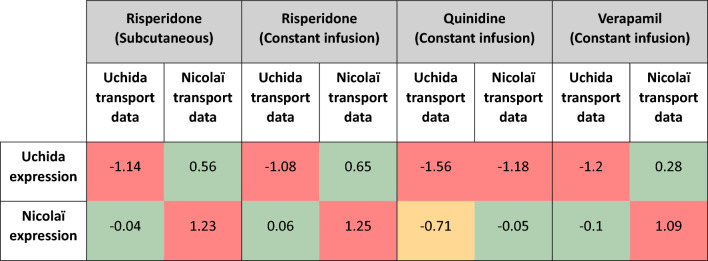
Median prediction errors of risperidone, quinidine and verapamil predictions made using LLC-PK1-mdr1a transport data from Uchida et al. [15] and Nicolaï et al. [16], using the LLC-PK1-mdr1a in vitro P-gp expression reported by Uchida et al. and by Nicolaï et al. PE values shaded in green, yellow and red fall within twofold error, threefold error and > threefold error, respectively

### Variability in in vitro derived parameters impacts the predicted rate and extent of distribution to the brain

**Fig. 8:**
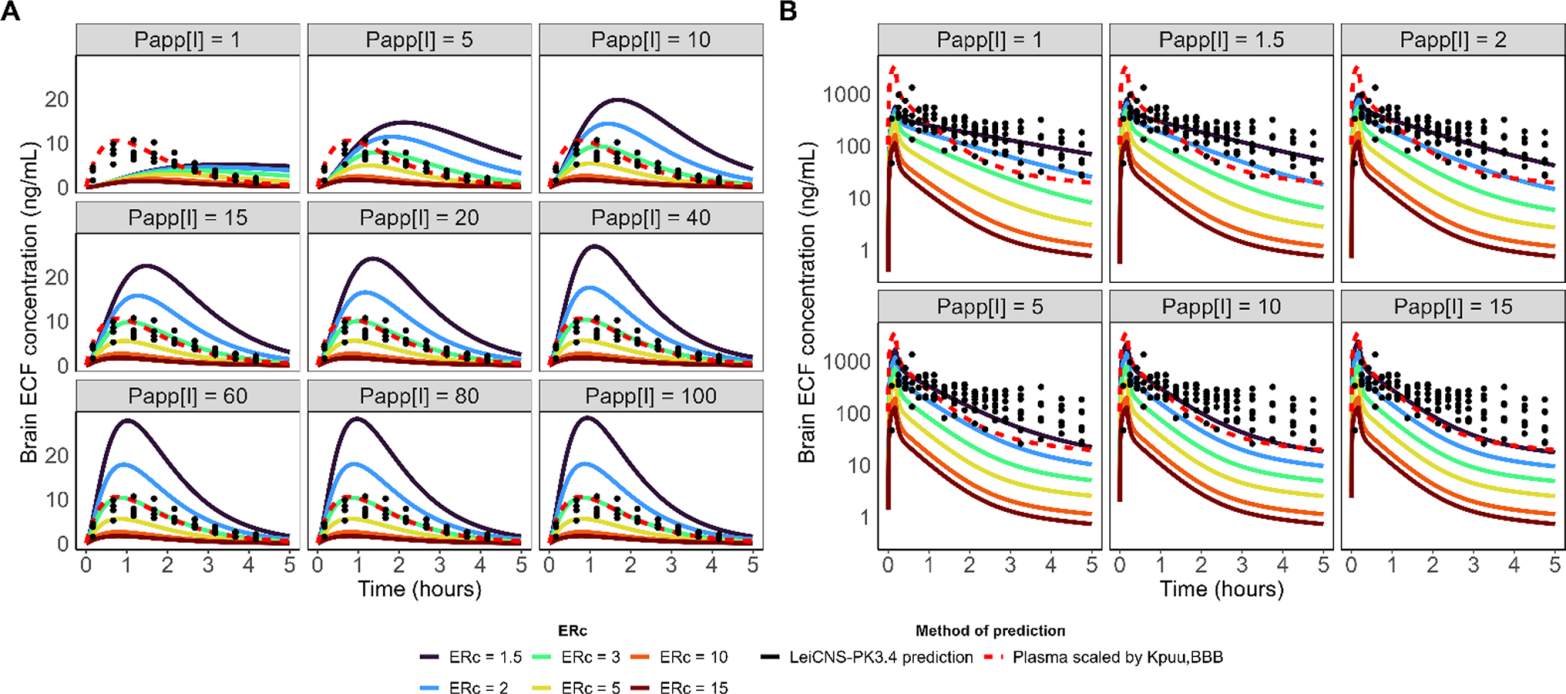
Impact of varying passive permeability (P_app_[I]) and ER_c_ on the predicted brainECF PK profiles of risperidone and morphine. The passive permeability used as input into the LeiCNS-PK3.4 model is given in the headers, while the ER_c_ used as input is indicated by the colour of the line. **A** Risperidone simulations are given using in vitro P-gp expression of 13.1 fmol/µg protein as reported in LLC-PK1-MDR1 cells. The dashed red line represents the unbound plasma concentration profile scaled by the observed Kp_uu,BBB_ of 0.147 (i.e., C_ECF_ = C_plasma,u_ * Kp_uu,BBB_). **B** Morphine simulations are given using the average in vitro P-gp expression in Caco-2 cells of 4.7 fmol/µg protein. The dashed red line represents the unbound plasma concentration profile scaled by the observed Kp_uu,BBB_ of 0.23. For both subplots, P_app_[I] values are given in 10^–6^ cm/s. Black dots are observed microdialysis data. The LeiCNS-PK3.4 simulations are shown as continuous lines. P_app_[I] here represents P_app,A:B_[I] (Color figure online)

To appreciate the impact of the variability in P_app.A:B_[I] and ER_c_ on the predicted rates and extent of drug distribution, we simulated the subcutaneous administration of risperidone and short infusion of morphine over a range of input values (Fig. 8). The LeiCNS-PK3.4 simulations are shown as continuous lines. The predicted profile that is obtained by only scaling the plasma PK profile by the observed Kp_uu,BBB_ (extent of distribution) is shown as a dashed, red line. For risperidone, the scaled plasma profile shows a good match with the extent of risperidone distribution, but overpredicts the rate of distribution (Fig. 8A). The morphine prediction using only the Kp_uu,BBB_ to scale the plasma profile misrepresents the observed rate of distribution in the brain ECF (Fig. 8B). The LeiCNS-PK3.4 simulations highlight that increasing passive permeability shifts the predicted rates of distribution to follow the shape of the plasma profile more closely. Variability in the ER_c_ mostly impacts the predicted extent of distribution. However, especially for risperidone at lower (< 20 * 10^–6^ cm/s) passive diffusion speed, increasing ER_c_ also impacts the predicted T_max_ (Fig. 8A). The impact of variability in passive permeability on the predicted rate of distribution for passively diffusing compounds shows similar behaviour, with high diffusion speeds approaching the shape of the plasma profile (Supplementary Fig. 4).

## Discussion

Transport data (e.g., P_app_[I] and ER_c_) for many drugs are widely available in literature, and have shown promise for prediction of P-gp mediated drug distribution across the BBB. An important distinction should be made here between predicting extent of distribution (i.e., the ratio of unbound drug concentration between brain ECF and plasma at steady state, Kp_uu,BBB_) and rate (i.e., with what speed a drug crosses the BBB) [17]. PBPK models allow for prediction of temporal profiles (representing both rate and extent) based on in vitro information, without relying on in vivo studies. However, in vitro data are known to be variable, the impact of which had not yet been investigated for CNS PBPK modelling. Additionally, the suitability of this data for brainECF PK predictions of multiple drugs and dosing schemes is not yet fully characterized. We therefore evaluated the reliability of literature derived in vitro data for in vivo predictions of brainECF PK, by introducing an in vitro informed P-gp mediated clearance into the LeiCNS-PBPK model (LeiCNS-PK3.4).

### In vitro transport data are informative of the in vivo rate of passive distribution

The LeiCNS-PK3.4 model was able to accurately predict unbound brainECF PK in the absence of active processes. This is in agreement with previous CNS PBPK models, which showed the same description of passive diffusion (CL_passive_) to allow accurate predictions of CSF distribution of passively diffusing compounds [19, 71, 72]. The P_app,A:B_[I] reported in literature for the passively diffusing compounds were either only available from one source or did not vary greatly. Simulations of the passively diffusing drugs (Supplementary Fig. 4) however show that varying passive diffusion has a great impact on the predicted rate of distribution of the drugs. The fact that we were able to adequately predict the brainECF PK of these compounds using the currently applied values therefore show the translational value of the in vitro transport data to in vivo.

Nicolaï et al. had to reduce in vitro passive diffusion tenfold for accurate prediction of Kp_uu,brain_ [16]. Such a reduction in passive diffusion rate has great implications for the predictions of temporal PK, as shown by the risperidone simulations. The risperidone passive permeability reported by Cousein et al. for example was more than tenfold lower than other risperidone transport values [62], which resulted in a poor prediction of the rate of distribution (Fig. 5). This highlights the difference between prediction of Kp_uu,brain_ (extent) and temporal brain (ECF) PK profiles (both rate and extent). Accurate prediction of one does not guarantee an accurate prediction of the other. This was also shown by Storelli et al., who predicted Kp_uu,brain_ using in vitro data within twofold of the observed value, but subsequently underpredicted temporal PK profiles [73].

In the risperidone and morphine simulations (Fig. 8) we included a prediction based on scaling the plasma profile by the extent of distribution (Kp_uu,BBB_). This resulted in a predicted rate of distribution that did not follow the observed rate of distribution, which was especially clear for a slowly diffusing compound like morphine. This shows the importance of separately accounting for rate and extent when predicting CNS exposure. The simulations show that the interplay between P_app,A:B_[I] and ER_c_ is important in determining the final temporal PK profiles.

### Variability in vitro impacts the predicted rate and extent of drug distribution and therefore reliability of the model

Including P-gp mediated clearance (CL_Pgp_) based on literature-derived in vitro data showed promising predictions for most of the P-gp substrates. The values of in vitro-derived parameters are different between sources, which can lead to markedly different predicted extents of distribution for the P-gp substrates, most clearly seen for the continuous infusion dosing regimens (Fig. 5B) (see for example the LLC-PK1-mdr1a based risperidone predictions). The MDCKII-MDR1 derived transport data, which show a high degree of variability in P_app,A:B_[I] for quinidine, risperidone and verapamil, showed the least variability in ER_c_ of the different cell lines, leading to the most similar predicted extents of distribution between sources. The model simulations support the observation that variability in ER_c_ is the major factor in determining the predicted extent of distribution.

P_app,A:B_[I] was also found to be variable between sources, which has important implications for the predicted rate of distribution to the brain (Fig. 8, Supplementary Fig. 4). Our risperidone simulations show that P_app,A:B_[I] values higher than 20×10^–6^ cm/s mirror the shape of the plasma PK profile, rather than the observed rate of distribution in the brain ECF. The two LLC-PK1-mdr1a based risperidone predictions using the data from Uchida et al. and Nicolaï et al. (P_app,A:B_[I] = 96.3 × 10^–6^ cm/s and 66.2 × 10^–6^ cm/s, respectively) therefore overpredict the rate of distribution as observed in vivo. The in vitro data by Nagaya et al., Inoue et al. and Feng et al. seem to resemble risperidone’s PK in vivo more closely. The reason for why exactly these studies report such different values for P_app,A:B_[I] should be studied more thoroughly, in order to serve as a robust input for LeiCNS-PK3.4.

The morphine simulations (Fig. 8B) indicate a requirement of low P_app.A:B_[I] and low ER_c_ in order to accurately capture both rate and extent of distribution in vivo. This agrees with what is reported in vitro for morphine, allowing accurate predictions of morphine’s rate and extent of distribution by LeiCNS-PK3.4. This shows that in vitro data can indeed hold important mechanistic information on a P-gp substrate’s distribution behaviour in vivo. We observe however that the variability observed for both P_app,A:B_[I] and ER_c_ has substantial impact on the robustness of the predicted rates and extent, which should be kept in mind when using in vitro data, especially when it is derived from a single source and not compared to other articles.

### Accounting for P-gp expression in vitro

Most of the published transport data did not report P_app,A:B_[I] and ER_c_ in conjunction with in vitro P-gp expression. The P-gp expression in vitro was found to be highly variable in literature. Therefore, instead of using a single P-gp expression value, we made predictions for the bandwidth of reported P-gp expression values. From that we conclude that the REF greatly influences the extent of drug distribution, and thereby the prediction accuracy. This is in line with Fenneteau et al. who identified the scaling factor associated with P-gp expression to be a sensitive parameter in their PBPK model [74], as well as the results by Ball et al. who showed an important role of the REF (named RAF in their study) on the predicted brainECF profile [23]. Most of the transport data have a median PE within twofold error when combined with one or multiple of the reported P-gp expression levels (Table 5 and Supplementary Tables 4 and 5). This indicates that currently, as long as the underlying reasons for variability in P-gp expression data are unknown and the actual P-gp expression in vitro is not given, this bandwidth P-gp expression approach gives a good indication of the PK profile in vivo.

Uncertainty in in vitro P-gp expression and its effect on the prediction of intestinal permeability has been investigated by Harwood et al., who recommended that both expression and permeability should be measured in conjunction for accurate IVIVE [75]. This was echoed by Verscheijden et al. who had to estimate a scaling factor in their brain PBPK model, since a REF based on a literature-derived expression value did not adequately describe observed data [19]. Interestingly, for the cases in which we combined LLC-PK1-mdr1a transport values with in vitro P-gp expression that were reported in conjunction, we observed poor prediction accuracies (> threefold PE, Table 6). In fact, for LLC-PK1-mdr1a, using in vitro expression values reported by other studies gave better predictions for risperidone, quinidine and verapamil than using the in vitro expression level given in the same publication (Table 6). We will now discuss multiple ideas that might (partly) explain this observation.

### Are P-gp expression and its activity linearly related?

Uchida et al. consistently reported a higher ER_c_ than Nicolaï et al., even though Nicolaï et al. reported an in vitro P-gp expression 4-fold higher than Uchida et al. [15, 16]. Based on the assumed correlation between expression of P-gp and its functionality, we would expect the P-gp expression to also be lower for Nicolaï et al. This is also what the model indicates to be required for accurate predictions (Table 6). When Nicolaï et al. scaled only through a REF, they observed a poor prediction accuracy of the Kp_uu,brain_ which they tended to overpredict (our predictions using Nicolaï data similarly overpredict the observed data, Table 6) [16]. A tenfold reduction of passive permeability allowed for more accurate predictions in their study. Not considered by Nicolaï et al. (nor by our model) is the distinction between *total* P-gp and *efflux active* (or functional) P-gp in a cell line [76, 77]. Functional P-gp is expressed at or near the tips of microvilli, which can successfully expel a drug [76]. Drug expelled by P-gp at the sides of microvilli (‘inactive’ P-gp) however will promptly collide with the membranes of neighbouring villi and be reabsorbed. The amount of functional P-gp has been reported to be tenfold lower than the total amount of P-gp in Caco-2 cells [76]. LLC-PK1 cells also possess microvilli [78, 79], as such a discrepancy between total P-gp and functional P-gp might explain why the Nicolaï et al. data require lower in vitro expression levels in our model predictions. It does not explain why the Uchida et al. efflux ratios are so high, as the functional P-gp concentration would have to be higher than the total amount to justify the use of a higher expression level than reported.

Besides the distinction between total and active P-gp, we might also question our and others’ assumptions (1) that activity and expression of P-gp are linearly correlated, and (2) that this relationship is drug-independent. A linear relationship between expression and activity of P-gp in vitro has been reported for quinidine, verapamil and vinblastine [40, 41]. This has also been reported for BCRP and OATP1B1 when normalizing to Na^+^-K^+^ ATPase expression [80]. In vivo, changes in intestinal P-gp expression influenced uptake of P-gp substrates [81]. However, others were unable to correlate expression and functionality of P-gp. A study on the hCMEC/D3 BBB cell line showed that an increase in both mRNA and protein expression of P-gp did not influence transport of rhodamine-123, a strong P-gp substrate [82]. Similarly, Kosztyu et al. were not able to relate P-gp expression (either protein or mRNA) to its activity in vitro [83]. Expression of P-gp on lymphocytes also did not show a relationship to functionality [84, 85], and an in vivo study showed that changes in P-gp protein expression at the rat BBB did not influence quinidine efflux [86]. At the dog BBB, individual differences in P-gp and BCRP expression did not correlate with the K_p,brain_ of quinidine and apafant (P-gp substrates) or dantrolene and daidzein (BCRP substrates) [87].

In the linear correlations reported by Tachibana et al., a change in expression did not yield the same change in maximum P-gp activity (V_max_) for different drugs [40]. Sanchez-Dengra et al. developed a PBPK model to predict brainECF PK, and fit empirical scaling factors for their IVIVE approach. They obtained different estimates depending on the drug, and correlated the scaling factors in a non-linear fashion with logP [88]. A drug-dependency of the scaling factor can also be observed in the current study. We first observed that a single scaling factor as applied in the current methodology is not generically applicable across drugs (Table 5 and Fig. 7). Moreover, a comparison of the PEs of the continuous infusion predictions made using the low in vitro P-gp expression for references that reported transport values for multiple drugs, showed a trend of PE_paliperidone_ > PE_risperidone_ > PE_verapamil_ > PE_quinidine_ (supplementary Fig. 3). If differences in P-gp activity between in vitro and in vivo would only depend on P-gp expression, a single scaling factor should give similar prediction errors for data retrieved from a single experimental setup. Overall, the current results are in line with other reports which suggest that the relationship between expression and activity of P-gp is not straightforward, at least at the BBB. Considering drug-specific differences together with differences in P-gp expression and morphology of the cell line(s) might therefore prove important for successful and robust IVIVE of CL_Pgp_ at the BBB.

### Methodological considerations

To account for the effect of other (endogenous) transporters present in vitro, we made use of the corrected efflux ratio (ER_c_). This was done by dividing the ER (obtained in the P-gp expressing cell line) by the ER[I], which is obtained though chemical inhibition of P-gp or by measuring in the parental cell line that does not overexpress P-gp. Chemical inhibitors that were used in the in vitro studies are Cyclosporine A, GF120918, verapamil and GW918. Cyclosporine A, GF120918 and verapamil all also inhibit BCRP and MRP1 [89]. Parental cell lines also are not perfect copies of the transfected cell line, as transporter expression might differ [90]. As such, it is likely that the currently calculated ER_c_ does not purely represent P-gp activity if a drug is a substrate for multiple transporters. For future research, it is recommend to investigate whether using a P-gp specific inhibitor [91] to determine ER[I] might be more reliable for a description of purely P-gp.

Another aspect that might improve the method is to use pmol transporter per gram (pmol/g) wet tissue weight as a measure of expression, instead of fmol P-gp/µg total protein [15, 39, 92]. This unit reduces inter-laboratory variability in transporter expression, as it accounts for differences in sample preparations and purity of the determined protein amounts [93]. An approach to convert fmol/µg protein to pmol/g wet tissue has been proposed [94, 95], however, this requires substantial knowledge of the experimental setup, which is not easily extracted from literature. Additionally, in vitro P-gp expression has not yet been reported in this unit, which restricted the current study to use of fmol/µg.

We did not limit the in vitro studies to those that use the rat homologue of P-gp, but also included mouse and human derived P-gp. We did not think this would significantly influence the rat brainECF PK predictions, as the rat, mouse and human homologues of P-gp share a highly conserved amino acid sequence (sequence similarity of 92% mouse-human, 97% mouse-rat) [96]. Additionally, others previously reported similar binding characteristics on a molecular level [96], a good correlation in mouse and rat ER in vitro [97], similar functionality in vivo of rat and mouse P-gp [98], and a good correlation between human and mouse P-gp ER (R^2^ = 0.92, *n* = 3300) [53]. Moreover, our model predictions do not show an inability of one of the homologues to accurately predict rat brainECF PK.

### Quinidine influx and differences between short infusion and continuous infusion dosing

The final point we would like to discuss is the difference between the short infusion (S.I.) and continuous infusion (C.I.) data of quinidine. The S.I. quinidine data was reported by Westerhout et al. [44]. P-gp was functional in this study, as coadministration of the P-gp inhibitor tariquidar significantly increased the Kp_uu,brain_ from 1.5 to 8.6. Therefore, these results indicate significant P-gp activity but also a role for influx transport of quinidine in vivo (as Kp_uu,brain_ > 1) that is not (sufficiently) present in vitro. This is supported by other reports that have indicated quinidine to be influxed by an influx transporter (like OATP1 or OATP2) and/or to inhibit influx of other compounds [99–102]. Ishida et al. have reported quinidine to be influxed during in vitro experiments in the Caco-2 cell line [103]. Quinidine is an example of how our model can highlight important physiological aspects in vivo that might be missed in vitro. Of note is that the influx led to great underprediction of the S.I. data (± 100-fold), but not of the C.I. data (overall underprediction between ± threefold and ± sixfold), giving predictions within threefold PE for all transport data when using the high in vitro expression. The extent of drug distribution varies between different brain regions [104]. This might play a role as the C.I. dataset measured ECF data in the hippocampus and frontal cortex, while the S.I. dataset measured in the striatum. The difference in rat type might also play a role as the C.I. dataset used Sprague–Dawley rats [47] while the S.I. dosing used Wistar rats [44]. Finally, the difference in the administration itself (C.I. versus S.I.) might influence the BBB transport kinetics. Overall, the reason for the difference in the C.I. and S.I. quinidine (and also paliperidone) datasets is unclear and warrants further investigation. Estimation of the influx together with P-gp transport [21] might allow for these aspects to be computationally studied.

## Conclusions

This study highlights that accurate rat brainECF PK predictions of passively diffusing drugs and P-gp substrates are possible by informing a PBPK model with in vitro data obtained from literature. Especially predictions based on data from MDCKII-MDR1 cells showed a high degree of agreement in the predicted extent of distribution between different studies, due to little variation in reported ER_c_ of drugs. In general, variability in ER_c_ strongly affects predicted extents of distribution. Variability in P_app,A:B_[I] was substantial (also within cell lines), which has important implications for the predicted rate of distribution. Large variability in the reported in vitro P-gp expression influenced robustness of the model predictions and confidence in the predictions when using a single value. Instead, bandwidth predictions based on the extremes of reported in vitro P-gp expression allowed a brainECF PK prediction area, giving a good indication about what to expect in vivo. However, whenever transport data and in vitro P-gp expression reported by a single study were used as input together, this did not guarantee an accurate prediction. Scaling in vitro CL_Pgp_ to in vivo only through differences in P-gp expression might as such not completely capture the differences in functionality. Important mechanistic information about the relationship between P-gp expression and functionality appears to be missing for robust scaling of P-gp activity at the BBB. Looking beyond just expression differences and considering other (drug-specific) factors might therefore improve the robustness of the IVIVE approach.

## Supplementary Information

Below is the link to the electronic supplementary material.

Supplementary file1 (DOCX 1774 KB)

## Data Availability

All data supporting the results in this study can be found within the main text and Supplementary Materials.
