# Supplementary material for: Reliability of in vitro data for the mechanistic prediction of brain extracellular fluid pharmacokinetics of P-glycoprotein substrates in vivo; are we scaling correctly?
Source: J Pharmacokinet Pharmacodyn. 2025 Feb 8;52(2):16. doi: 10.1007/s10928-025-09963-w (PMC11807079; doi:10.1007/s10928-025-09963-w)
Supplement: Supplementary file 1 — Supplementary file1 (DOCX 1774 KB) [file 10928_2025_9963_MOESM1_ESM.docx]

**Supplementary materials to:**

Reliability of *in vitro* data for the mechanistic prediction of brain extracellular fluid pharmacokinetics of P-glycoprotein substrates *in vivo*; are we scaling correctly?
Authors: Daan W. van Valkengoed^1^, Makoto Hirasawa^1^, Vivi Rottschäfer^2,3^, Elizabeth C.M. de Lange^1*^

**Affiliations:**
1: Division of Systems Pharmacology and Pharmacy, Leiden University, The Netherlands
2: Mathematical Institute, Leiden University, The Netherlands
3: Korteweg-de Vries Institute for Mathematics, University of Amsterdam, The Netherlands

*Corresponding author (email: [ecmdelange@lacdr.leidenuniv.nl](mailto:ecmdelange@lacdr.leidenuniv.nl))
Gorlaeus Laboratories, Einsteinweg 55, 2333 CC Leiden, The Netherlands
Tel : +31 71 527 6330

**Supplementary table 1: Drug physicochemical properties used for all model predictions.** All values were taken from Drug bank version 5.1.12, except for verapamil which were taken from PubChem. All f_up_ values were derived from Yamamoto et al. (2017)^1^, except for verapamil which was reported by Holt et al. (2019)^2^. * Morphine pKa and pKb values were taken from Kauffman et al. (1975) ^3^

| **Drug** | **Molecular weight** | **logP** | **pKa** | **pKb** | **f_up_** |
| --- | --- | --- | --- | --- | --- |
| Acetaminophen | 151.2 | 0.51 | 9.46 | -4.4 | 0.81 |
| Morphine | 285.3 | 0.99 | 9.63* | 7.93^*^ | 0.83 |
| Paliperidone | 426.5 | 2.3 | 13.74 | 8.76 | 0.08 |
| Quinidine | 324.2 | 2.82 | 13.89 | 9.05 | 0.14 |
| Raclopride | 347.2 | 3.19 | 6.26 | 8.47 | 0.07 |
| Risperidone | 410.5 | 3.27 | NA | 8.76 | 0.07 |
| Verapamil | 454.6 | 3.8 | NA | 9.07 | 0.09 |

| Parameter category (units) | Name (abbreviation) | Value |
| --- | --- | --- |
| Volumes (mL) | Total brain (V_brain_) | 1.88 |
|  | Brain ECF (V_ECF_) | 0.36 |
|  | Brain cell lysosome (V_LYS_) | 0.018 |
|  | Lateral ventricles (V_LV_) | 0.0075 |
|  | Third/fourth ventricles (V_TFV_) | 0.0075 |
|  | Cisterna magna (V_CM_) | 0.017 |
|  | Subarachnoid space (V_SAS_) | 0.135 |
|  | Microvasculature (V_MV_) | 0.054 |
| Flows (mL/min) | ECF bulk flow (Q_ECF_) | 0.0002 |
|  | CSF (Q_CSF_) | 0.0022 |
|  | Cerebral blood flow (Q_CBF_) | 2.87 |
| Surface areas (cm^2^) | Surface area of BBB (SA_BBB_) | 155 |
|  | Surface area of BCSFB (SA_BCSFB_) | 25 |
|  | Surface area of brain cell membrane (SA_BCM_) | 4250 |
|  | Surface area of lysosomes (SA_LYS_) | 2700 |
| pH | pH microvasculature (pH_MV_) | 7.4 |
|  | pH brain ECF (pH_ECF_) | 7.3 |
|  | pH brain ICF (pH_ICF)_ | 7 |
|  | pH lysosomes (pH_LYS_) | 5 |
|  | pH CSF (pH_CSF_) | 7.3 |
| Effective surface area (%) | Transcellular BBB transport (SA_fraction.BBB.trans_) | 99.8 |
|  | Paracellular BBB transport (SA_fraction.BBB.para_) | 0.006 |
|  | Transcellular BCSFB transport (SA_fraction.BCSFB.trans_) | 99.8 |
|  | Paracellular BCSFB transport (SA_fraction.BCSFB.para_) | 0.05 |
| Weight (kg) | Weight (WT) | 0.25 |
| Brain phospholipid fraction (unitless) | Brain phospholipids volume fraction (V_phb_) | 0.0533 |

**Supplementary table 2:** **Rat physiological parameters used for all model predictions.** Taken from Saleh et al. (2021)^4^.

| System | P-gp expression  (fmol/µg protein) | Reference |
| --- | --- | --- |
| Rat | 19.65 | Al Feteisi (2018)^5^ |
| Rat | 19.1 | Hoshi (2013)^6^ |
| Caco-2 | 7.89 | Miliotis (2011)^7^ |
| Caco-2 | 6.92 | Harwood (2016)^8^ |
| Caco-2 | 2.0 | Harwood (2016)^8^ |
| LLC-PK1-MDR1 | 13.1 | Nicolaï (2020)^9^ |
| LLC-PK1-mdr1a | 61.0 | Nicolaï (2020)^9^ |
| LLC-PK1-mdr1a | 15.2 | Uchida (2011)^10^ |
| MDCKII-MDR1 | 10.3 | Bao (2019)^11^ |
| MDCKII-MDR1 | 2.08 | Feng (2019)^12^ |
| MDCKII-MDR1 | 1.9 | Di (2011)^13^ |
| MDCKII-MDR1 | 2.847 | Jacqueroux (2020)^14^ |
| MDCKII-MDR1 | 2.394 | Jacqueroux (2020)^14^ |

**Supplementary table 3: P-gp protein expression in fmol/µg protein for *in vitro* and *in vivo* systems as reported in literature**


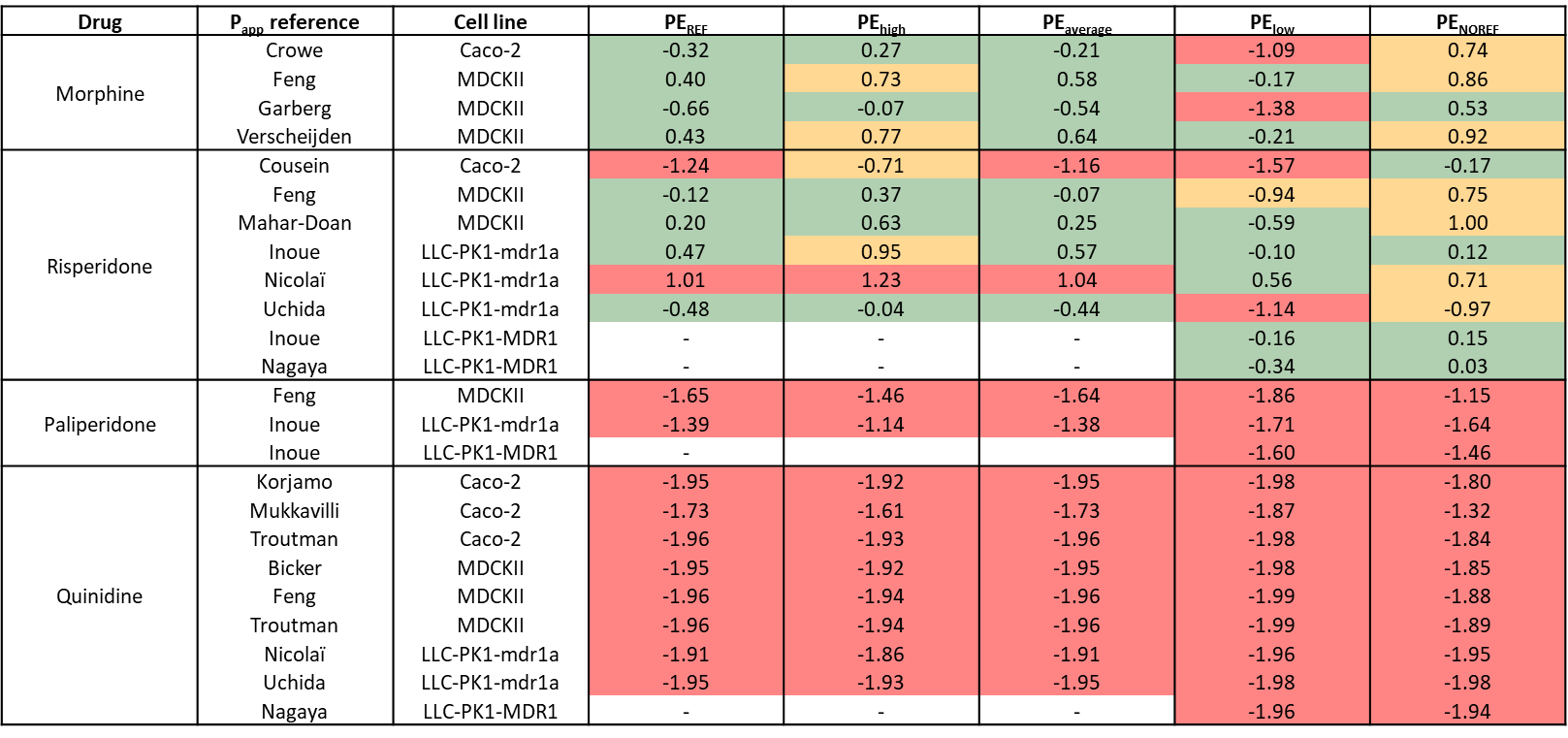
**Supplementary table 4: Overview of median prediction errors (PE) for all possible short infusion predictions.** PE_REF_ corresponds to the PE based on the entire bandwidth of predictions, while PE_high_, PE_average_, and PE_low_ are the PE observed for the predictions using the highest, average and lowest *in vitro* P-gp expression, respectively. PE_NOREF_ is the PE of the prediction made without a REF. PE values shaded in green, orange and red fall within 2-fold error, 3-fold error and >3-fold error, respectively.


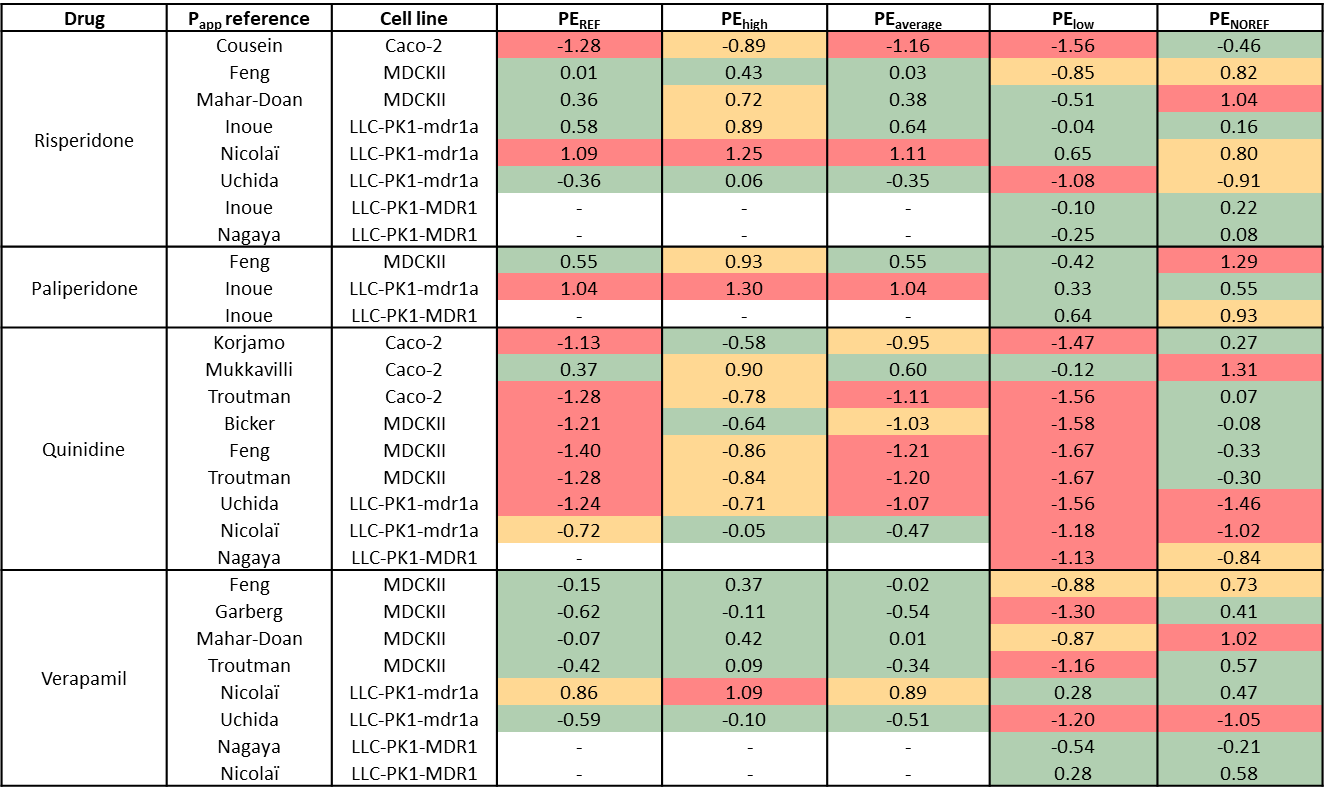
**Supplementary table 5: Overview of median prediction errors (PE) for all possible continuous infusion predictions.** PE_REF_ corresponds to the PE based on the entire bandwidth of predictions, while PE_high_, PE_average_, and PE_low_ are the PE observed for the predictions using the highest, average and lowest *in vitro* P-gp expression, respectively. PE_NOREF_ is the PE of the prediction made without a REF. PE values shaded in green, orange and red fall within 2-fold error, 3-fold error and >3-fold error, respectively.


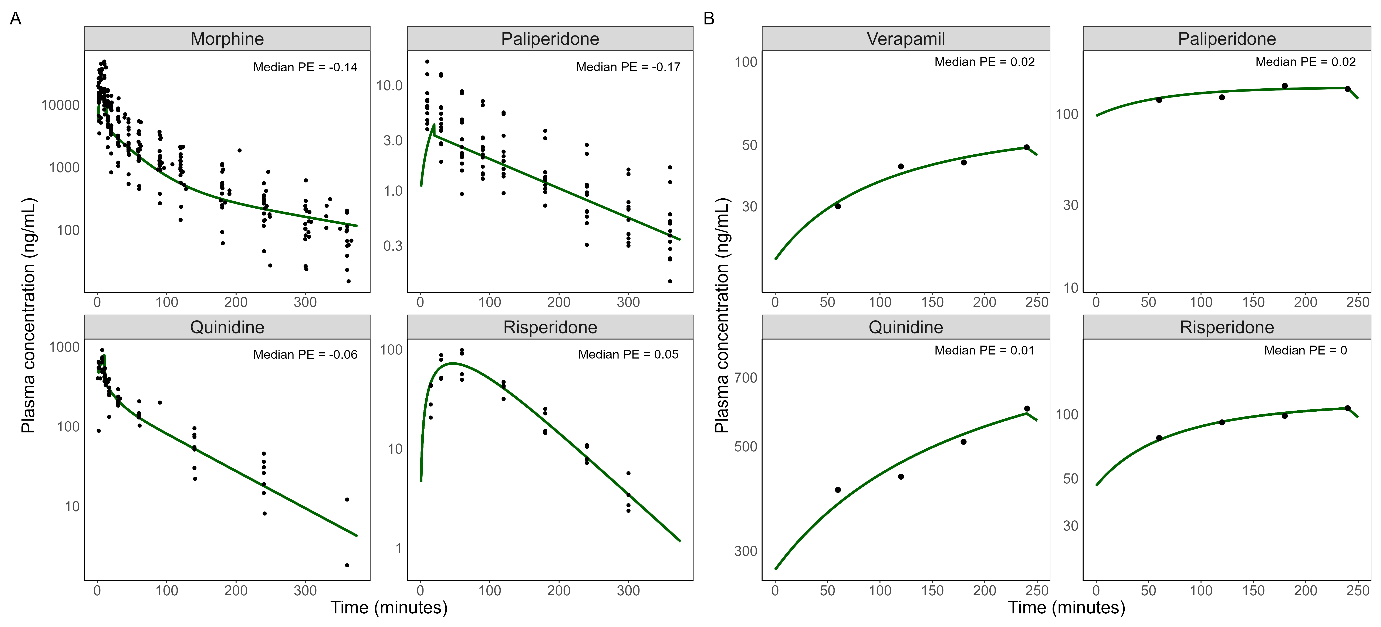


**Supplementary figure 1: Plasma PK predictions of the short infusion (A) and continuous infusion (B) dosing regimens.** All plasma data concerned unbound measurements, except for morphine which are total concentrations.


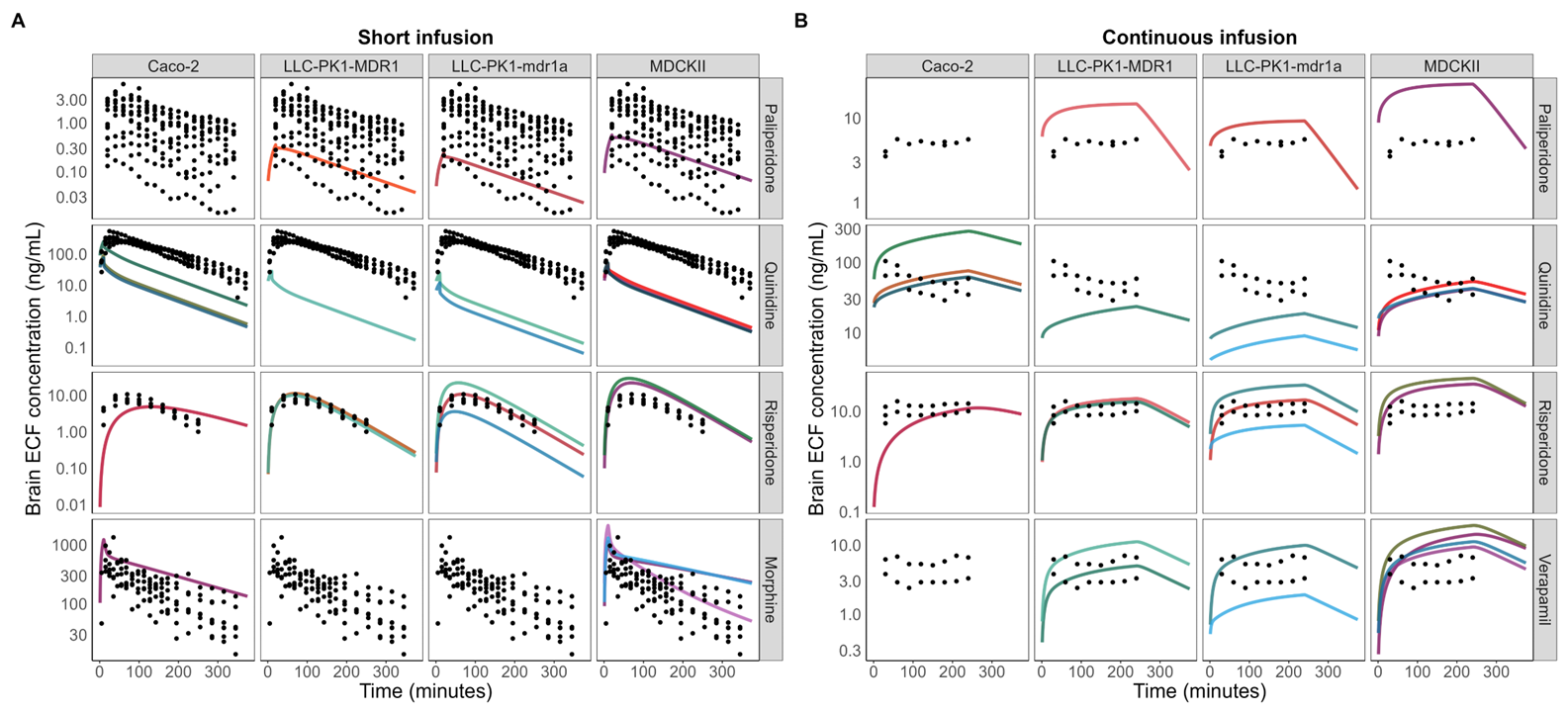

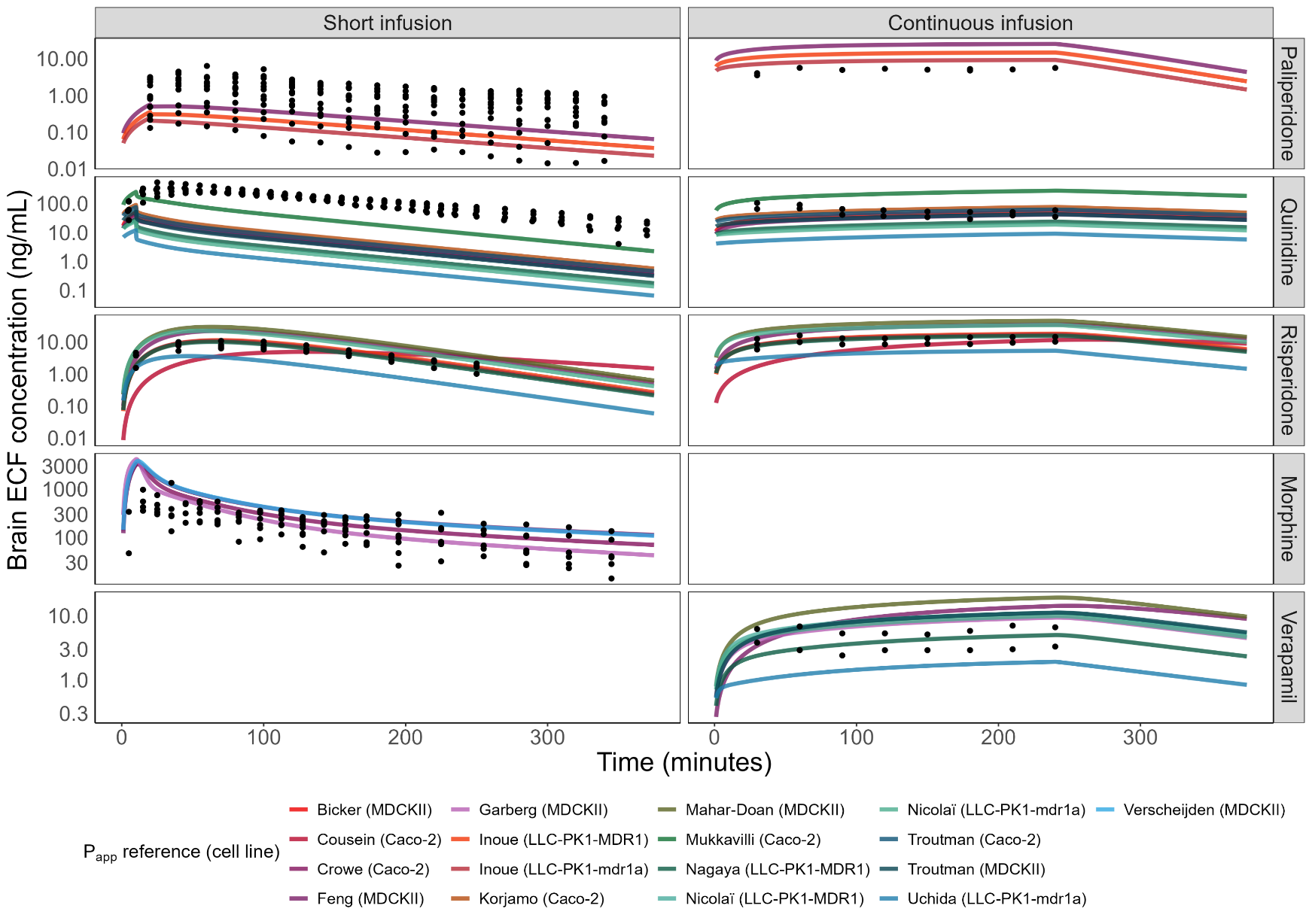


**Supplementary figure 2:** **Rat brainECF PK predictions after (A) short infusion (or subcutaneous dose administration) and (B) continuous infusion of the P-gp substrates paliperidone, quinidine, risperidone, morphine and verapamil without scaling for differences in P-gp protein expression.** Predictions are shown as coloured lines. Reference of the P_app_ values corresponds to the colour of the predicted line. Each column shows which cell line was used to determine P_app_ and ER_c_ values used as input for the prediction. Each row indicates a different drug. Plots without predictions indicate a lack of transport data in a cell line for a given drug. Observed unbound brainECF concentrations are shown as black points.

##
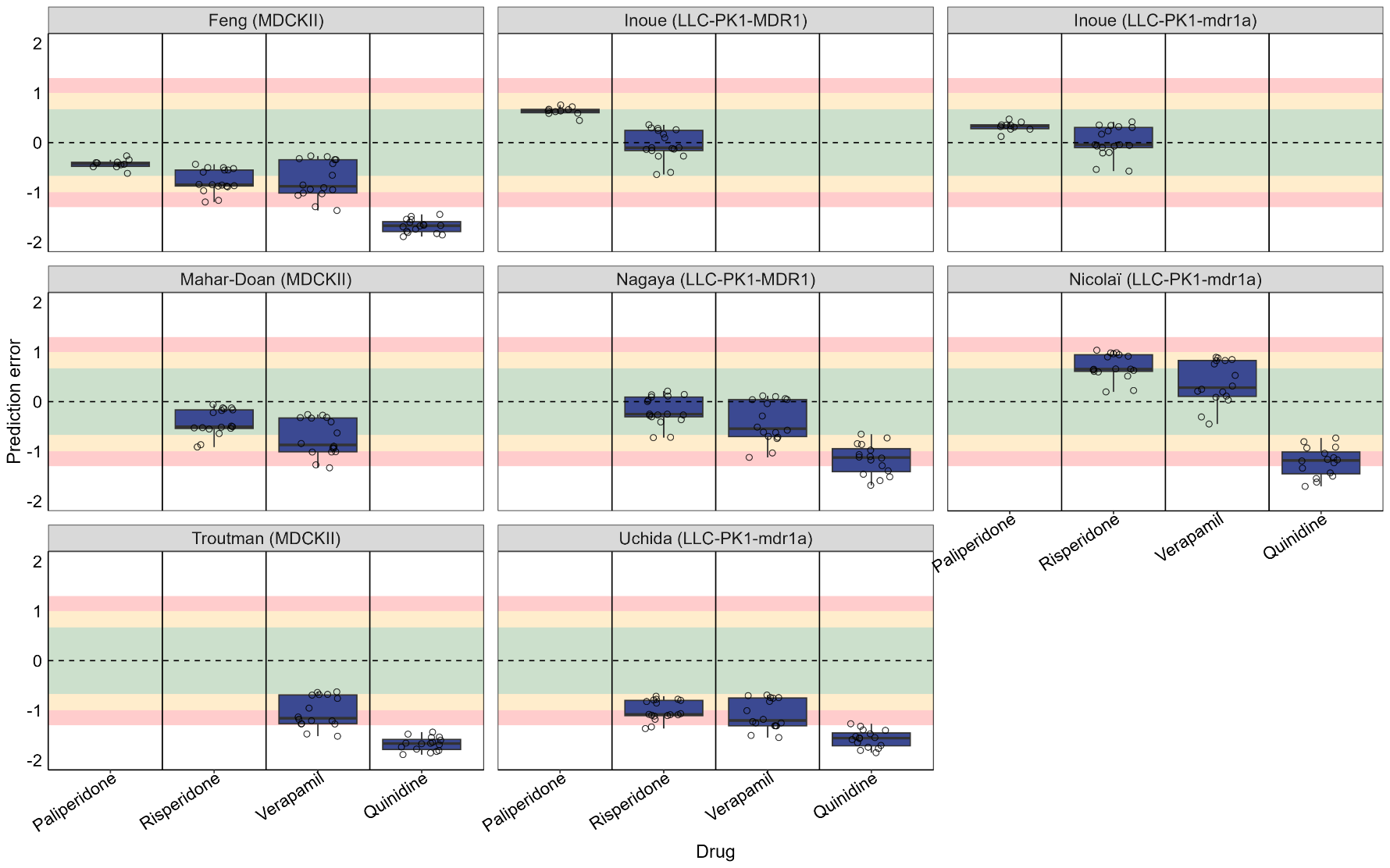


**Supplementary figure 3:** **Prediction errors (PE) of the continuous infusion predictions for references that reported transport values for more than one drug**. Predictions were made using the lowest in vitro P-gp expression for each cell line. The areas corresponding to <2-, <3- and <5-fold over- and underprediction are highlighted in green, yellow, and red, respectively. The PE of 0 is indicated with a dashed line.


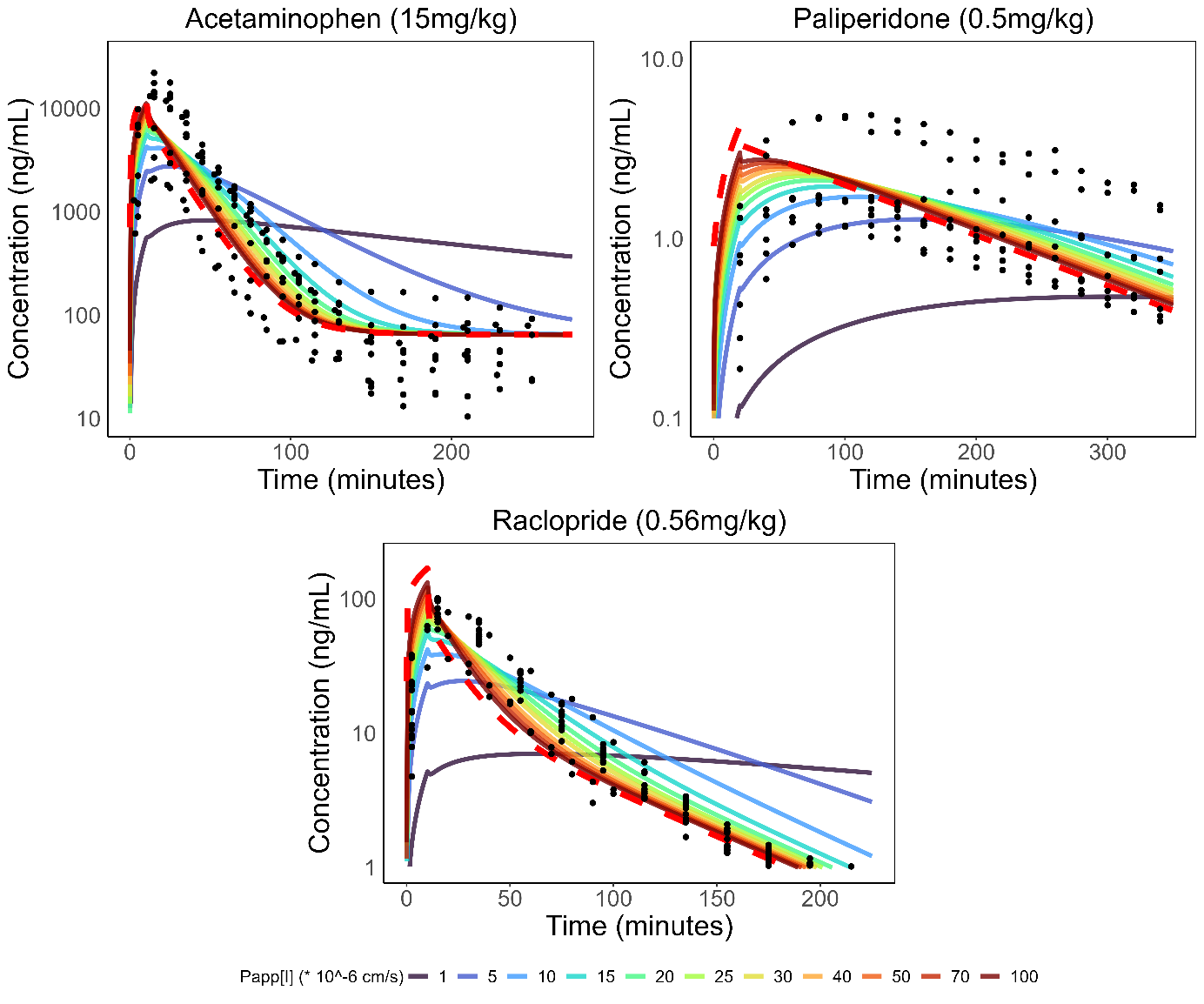


**Supplementary figure 4: Impact of different values of passive permeability (P_app,A:B_[I]) on the predicted brainECF PK profiles of the passively diffusing drugs acetaminophen, paliperidone (co-administered with tariquidar) and raclopride.** The coloured lines are LeiCNS-PK3.4 predictions using different values of P_app,A:B_[I] to calculate passive clearance across the blood-brain-border (BBB). The dashed red line represents the description of the plasma PK profile. Black dots represents observed microdialysis data in rat brainECF.

**References**

1 Yamamoto, Y. *et al.* Predicting Drug Concentration-Time Profiles in Multiple CNS Compartments Using a Comprehensive Physiologically-Based Pharmacokinetic Model. *CPT Pharmacometrics Syst Pharmacol* **6**, 765-777, doi:10.1002/psp4.12250 (2017).

2 Holt, K., Ye, M., Nagar, S. & Korzekwa, K. Prediction of Tissue-Plasma Partition Coefficients Using Microsomal Partitioning: Incorporation into Physiologically based Pharmacokinetic Models and Steady-State Volume of Distribution Predictions. *Drug Metab Dispos* **47**, 1050-1060, doi:10.1124/dmd.119.087973 (2019).

3 Kaufman, J. J., Semo, N. M. & Koski, W. S. Microelectrometric titration measurement of the pKa's and partition and drug distribution coefficients of narcotics and narcotic antagonists and their pH and temperature dependence. *J Med Chem* **18**, 647-655, doi:10.1021/jm00241a001 (1975).

4 Saleh, M. A. A., Loo, C. F., Elassaiss-Schaap, J. & De Lange, E. C. M. Lumbar cerebrospinal fluid-to-brain extracellular fluid surrogacy is context-specific: insights from LeiCNS-PK3.0 simulations. *J Pharmacokinet Pharmacodyn* **48**, 725-741, doi:10.1007/s10928-021-09768-7 (2021).

5 Al Feteisi, H. *et al.* Identification and quantification of blood-brain barrier transporters in isolated rat brain microvessels. *J Neurochem* **146**, 670-685, doi:10.1111/jnc.14446 (2018).

6 Hoshi, Y. *et al.* Quantitative atlas of blood-brain barrier transporters, receptors, and tight junction proteins in rats and common marmoset. *J Pharm Sci* **102**, 3343-3355, doi:10.1002/jps.23575 (2013).

7 Miliotis, T. *et al.* Development of a highly sensitive method using liquid chromatography-multiple reaction monitoring to quantify membrane P-glycoprotein in biological matrices and relationship to transport function. *Drug Metab Dispos* **39**, 2440-2449, doi:10.1124/dmd.111.040774 (2011).

8 Harwood, M. D. *et al.* In Vitro-In Vivo Extrapolation Scaling Factors for Intestinal P-Glycoprotein and Breast Cancer Resistance Protein: Part I: A Cross-Laboratory Comparison of Transporter-Protein Abundances and Relative Expression Factors in Human Intestine and Caco-2 Cells. *Drug Metab Dispos* **44**, 297-307, doi:10.1124/dmd.115.067371 (2016).

9 Nicolaï, J. *et al.* Impact of In Vitro Passive Permeability in a P-gp-transfected LLC-PK1 Model on the Prediction of the Rat and Human Unbound Brain-to-Plasma Concentration Ratio. *Pharm Res* **37**, 175, doi:10.1007/s11095-020-02867-z (2020).

10 Uchida, Y., Ohtsuki, S., Kamiie, J. & Terasaki, T. Blood-brain barrier (BBB) pharmacoproteomics: reconstruction of in vivo brain distribution of 11 P-glycoprotein substrates based on the BBB transporter protein concentration, in vitro intrinsic transport activity, and unbound fraction in plasma and brain in mice. *J Pharmacol Exp Ther* **339**, 579-588, doi:10.1124/jpet.111.184200 (2011).

11 Bao, X. *et al.* Protein Expression and Functional Relevance of Efflux and Uptake Drug Transporters at the Blood-Brain Barrier of Human Brain and Glioblastoma. *Clin Pharmacol Ther* **107**, 1116-1127, doi:10.1002/cpt.1710 (2020).

12 Feng, B. *et al.* Validation of Human MDR1-MDCK and BCRP-MDCK Cell Lines to Improve the Prediction of Brain Penetration. *J Pharm Sci* **108**, 2476-2483, doi:10.1016/j.xphs.2019.02.005 (2019).

13 Di, L. *et al.* Development of a new permeability assay using low-efflux MDCKII cells. *J Pharm Sci* **100**, 4974-4985, doi:10.1002/jps.22674 (2011).

14 Jacqueroux, E. *et al.* Value of quantifying ABC transporters by mass spectrometry and impact on in vitro-to-in vivo prediction of transporter-mediated drug-drug interactions of rivaroxaban. *Eur J Pharm Biopharm* **148**, 27-37, doi:10.1016/j.ejpb.2020.01.002 (2020).
